# Supplementary material for: Global phylogeny and taxonomy of Artemisia
Source: Nat Commun. 2025 Oct 8;16:8648. doi: 10.1038/s41467-025-64039-0 (PMC12508166; doi:10.1038/s41467-025-64039-0)
Supplement: Supplementary file 1 — Supplementary Information [file 41467_2025_64039_MOESM1_ESM.pdf]

Global phylogeny and taxonomy of *Artemisia*

Jiao *et al.*

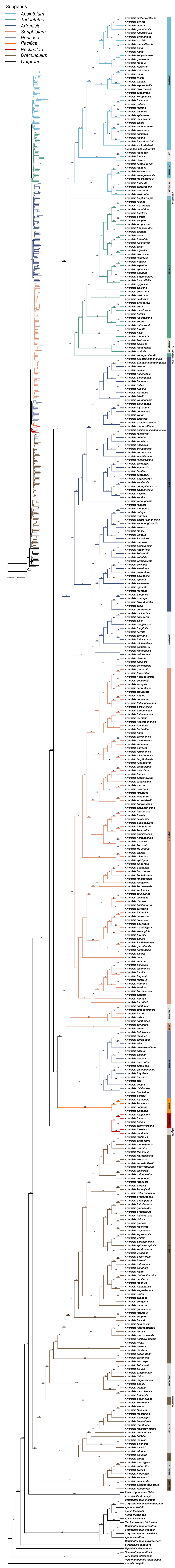

Supplementary Figure 1. Concatenated maximum likelihood (ML) tree of 414 species (394 *Artemisia* + 20 allied species) based on the gigamatrix (GM) dataset. Node supports were evaluated with 100 bootstrap replicates; values above branches denote bootstrap support (BS). Branch colors correspond to different subgenera, and colored bars indicate sections. Branch lengths are shown in the upper-left inset.

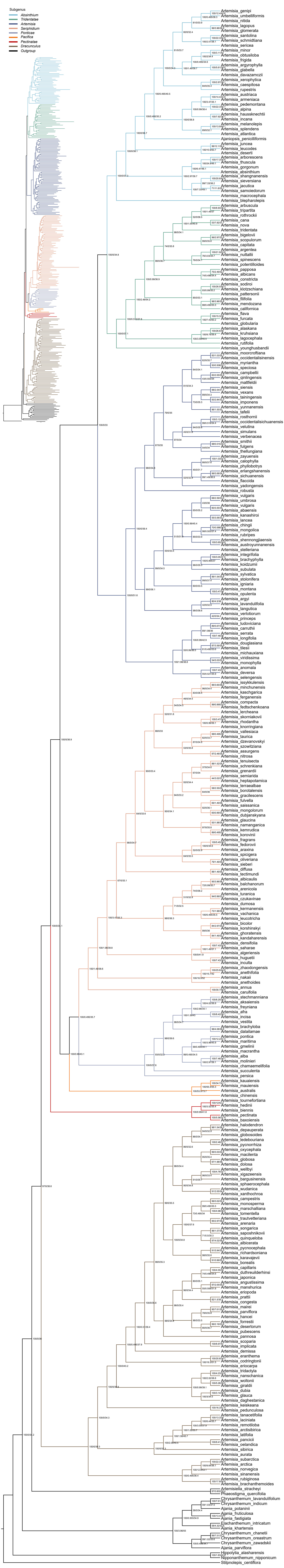

Supplementary Figure 2. Concatenated ML tree of 314 species (298 *Artemisia* + 16 allied species) based on 202 nuclear low-copy genes (NLC dataset). Node supports were assessed via 100 bootstrap replicates; values above branches denote bootstrap support (BS), gene concordance factor (gCF), and site concordance factor (sCF), respectively. Branch colors correspond to subgenera, and branch lengths are shown in the upper-left inset.

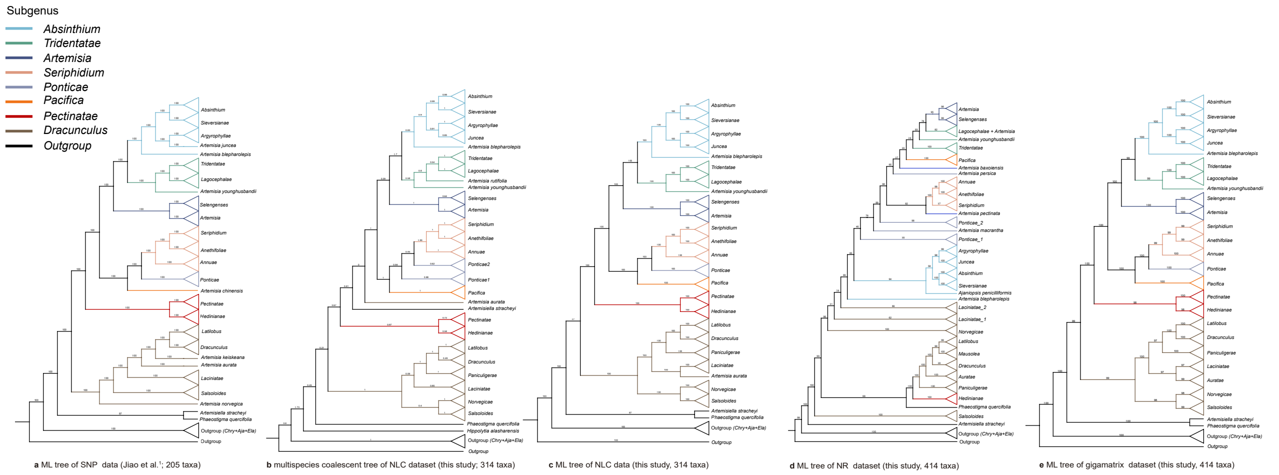

Supplementary Figure 3. Comparison of *Artemisia* phylogenies. a Simplified ML tree from Jiao et al.[1] using single nucleotide polymorphism (SNP) data. Bootstrap percentages from ML analysis are shown above branches. **b** Simplified multigene coalescent species tree inferred from 202 nuclear low-copy genes (NLC dataset). Local posterior probabilities are indicated behind nodes (see Supplementary Figure 4 for details). **c** Simplified ML tree of concatenated NLC dataset (Supplementary Figure 2). Bootstrap percentages from ML analysis are shown above branches. **d** Simplified ML tree of concatenated ribosomal nuclear DNA sequences (ITS + ETS; NR dataset). Bootstrap percentages from ML analysis are shown above branches (Supplementary Figure 5).
e Simplified ML tree of concatenated gigamatrix (GM dataset; Supplementary Figure 1). Bootstrap percentages from ML analysis are shown above branches. Branch colors correspond to *Artemisia* different subgenera of *Artemisia*. Tip names in simplified trees match section names in Figure 2.

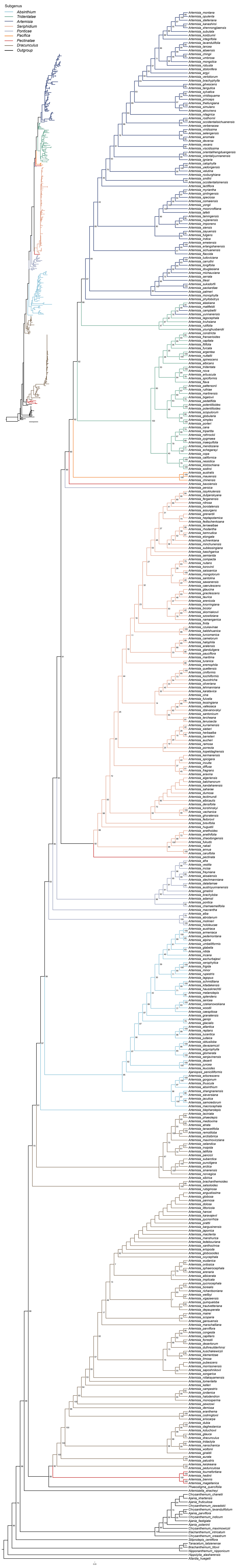

Supplementary Figure 4. Concatenated ML tree of 414 species (394 *Artemisia* + 20 allied species) based on ribosomal nuclear DNA sequences (ITS + ETS; NR dataset). Node supports were assessed via 100 bootstrap replicates; values above branches denote bootstrap support (BS). Branch colors correspond to subgenera, and branch lengths are shown in the upper-left inset.

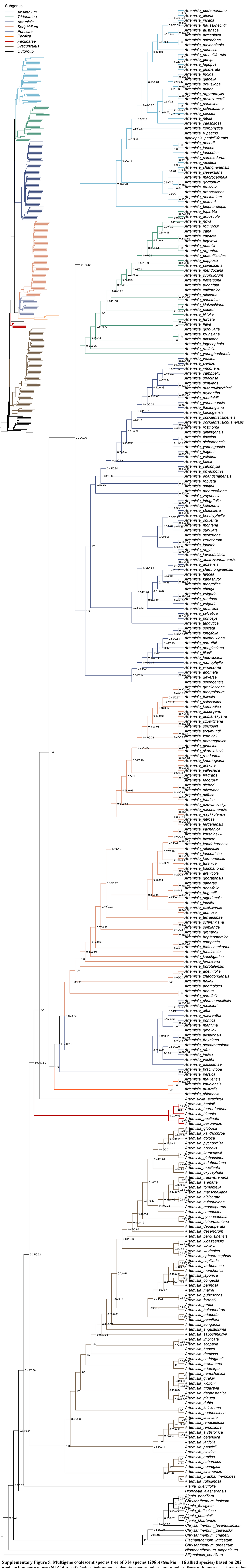

Supplementary Figure 5. Multigene coalescent species tree of 314 species (298 *Artemisia* + 16 allied species) based on 202 nuclear low-copy genes (NLC dataset). Values behind nodes denote support values and *p*-values from polytomy tests (two-tailed test), with the null hypothesis that branch lengths equal zero (implying the node should be a polytomy, p-value ≥ 0.1). Branch colors correspond to subgenera, and branch lengths are shown in the upper-left inset.

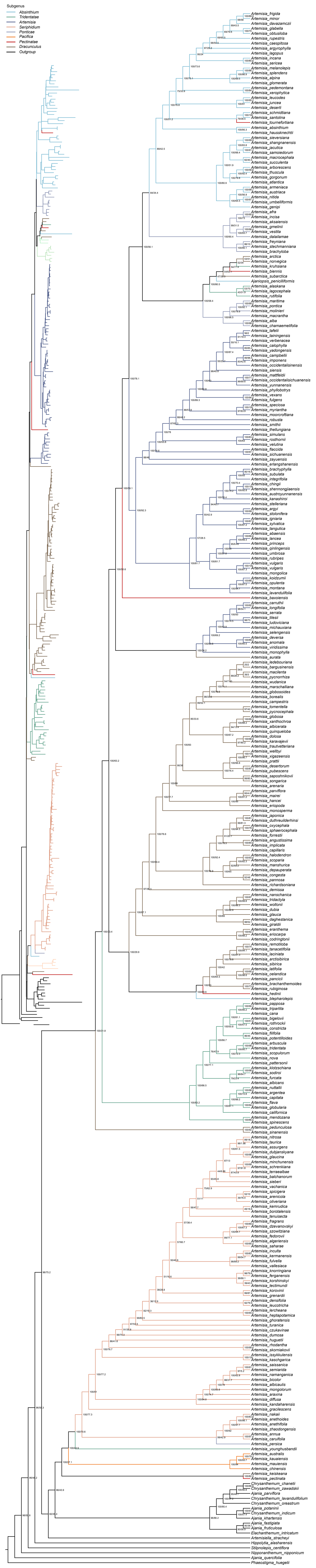

Supplementary Figure 6. Concatenated ML tree of 314 species (298 *Artemisia* + 16 allied species) based on complete plastid genomes (plastome dataset). Node supports were assessed via 100 bootstrap replicates; values above branches denote bootstrap support (BS) and site concordance factor (sCF), respectively. Branch colors correspond to subgenera, and branch lengths are shown in the upper-left inset.

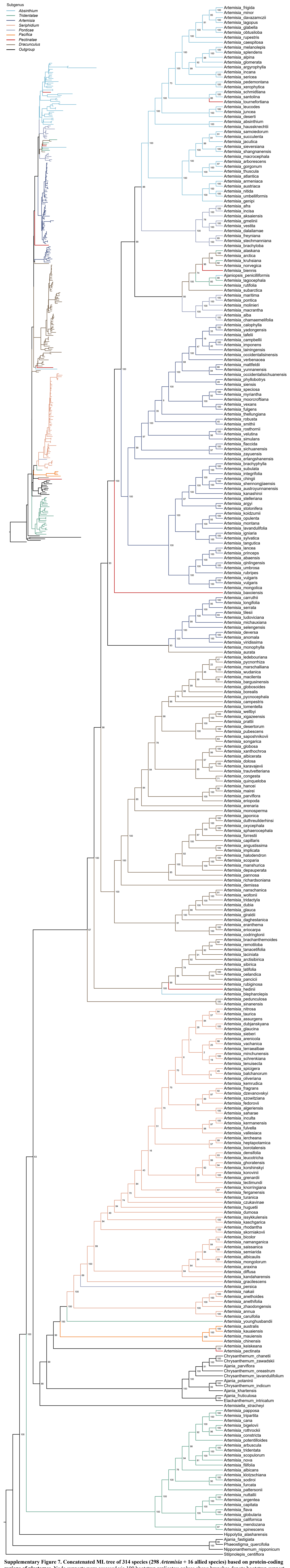

Supplementary Figure 7. Concatenated ML tree of 314 species (298 *Artemisia* + 16 allied species) based on protein-coding regions of plastomes. Node supports were assessed via 100 bootstrap replicates; values above branches denote bootstrap support (BS). Branch colors correspond to subgenera, and branch lengths are shown in the upper-left inset.

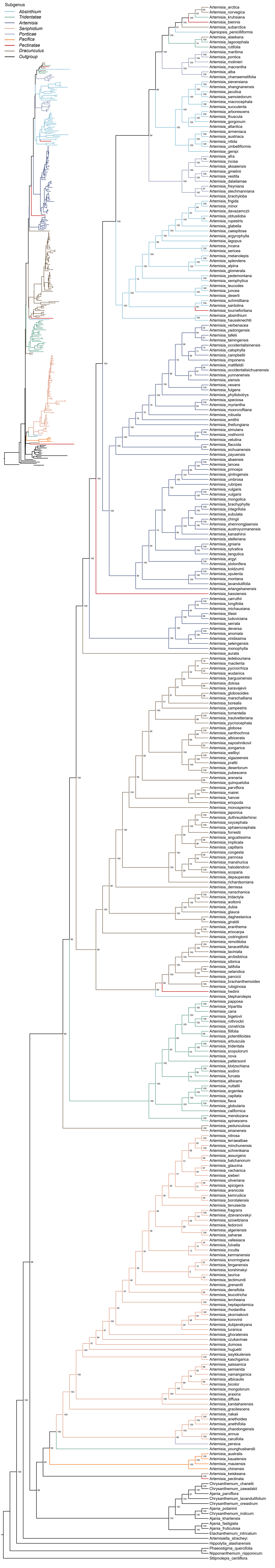

Supplementary Figure 8. Concatenated ML tree of 314 species (298 *Artemisia* + 16 allied species) based on non-coding regions of plastomes. Node supports were assessed via 100 bootstrap replicates; values above branches denote bootstrap support (BS). Branch colors correspond to subgenera, and branch lengths are shown in the upper-left inset.

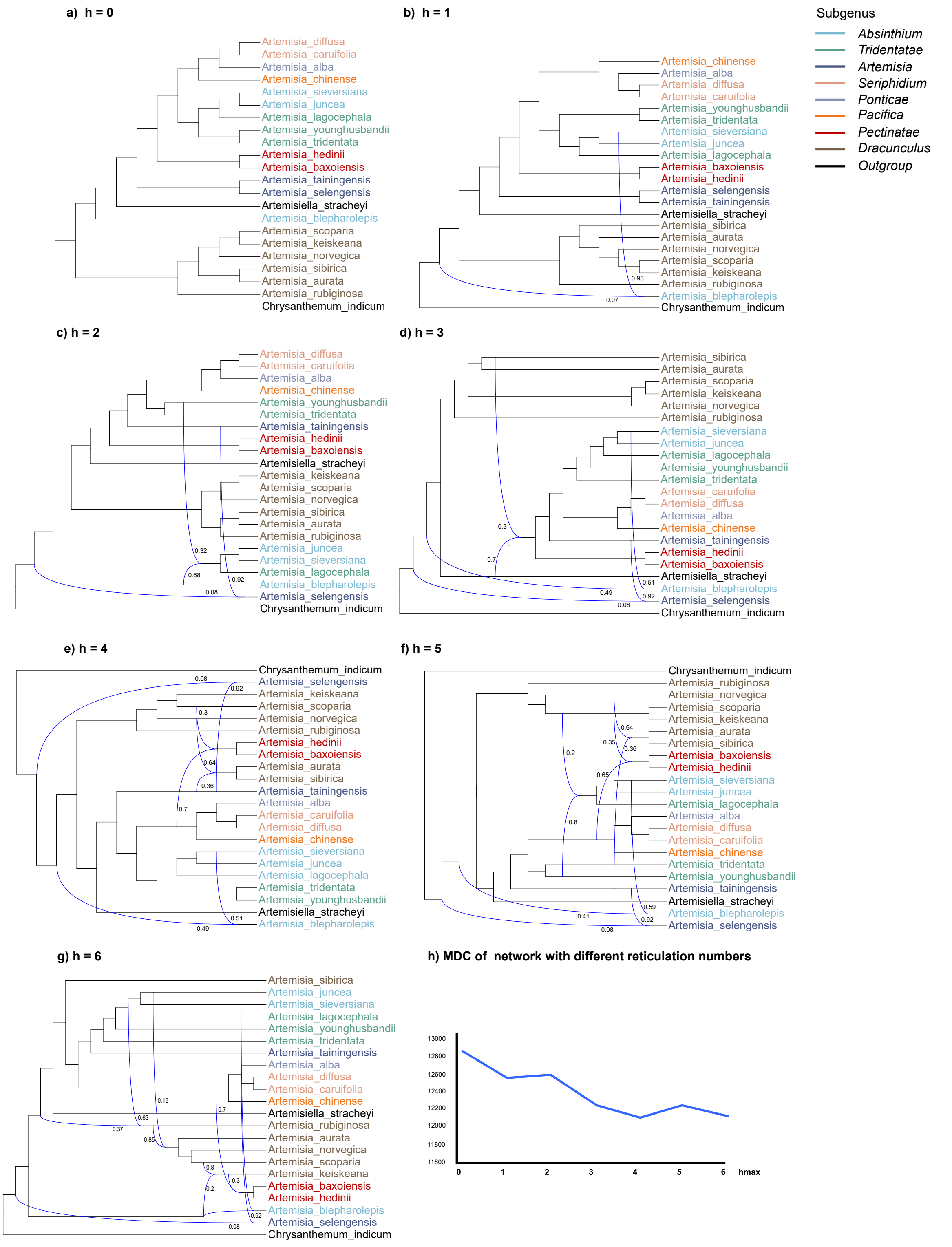

Supplementary Figure 9. Species network inferred from PhyloNet pseudolikelihood analyses based on 202 nuclear low-copy genes for 22 taxa (dataset 1), with 0 to 6 maximum reticulations (a–g). Blue curved branches denote potential hybridization events, and values indicate major/minor inheritance probabilities at hybrid nodes. Tip label colors correspond to subgenera. **h)** MDC (minimizing deep coalescence) of network with different reticulation numbers (the lower its value, the more optimal the network).

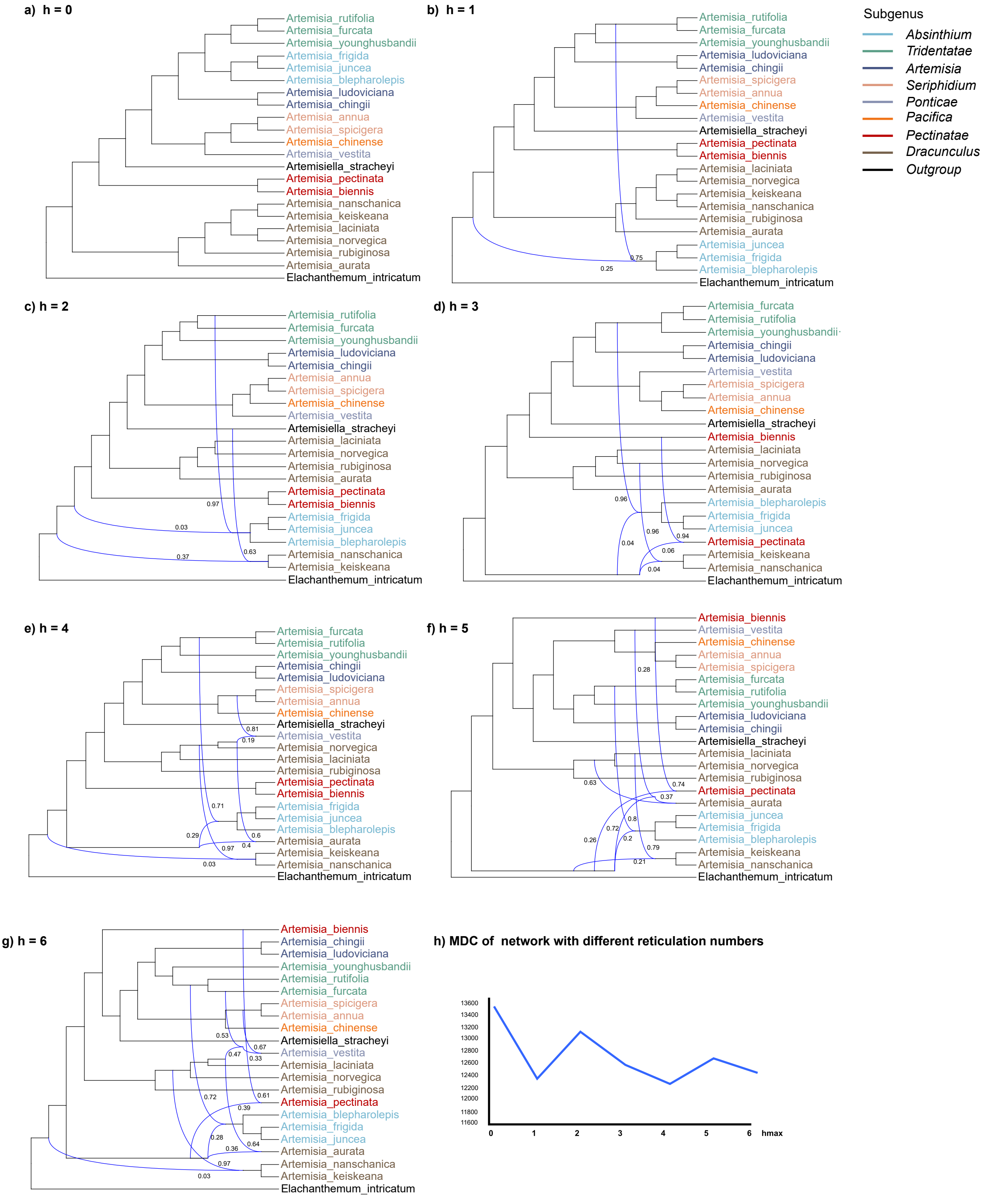

Supplementary Figure 10. Species network inferred from PhyloNet pseudolikelihood analyses based on 202 nuclear low-copy genes for 22 taxa (dataset 2), with 0 to 6 maximum reticulations (a–g). Blue curved branches denote potential hybridization events, and values indicate major/minor inheritance probabilities at hybrid nodes. Tip label colors correspond to subgenera. **h)** MDC (minimizing deep coalescence) of network with different reticulation numbers (the lower its value, the more optimal the network).

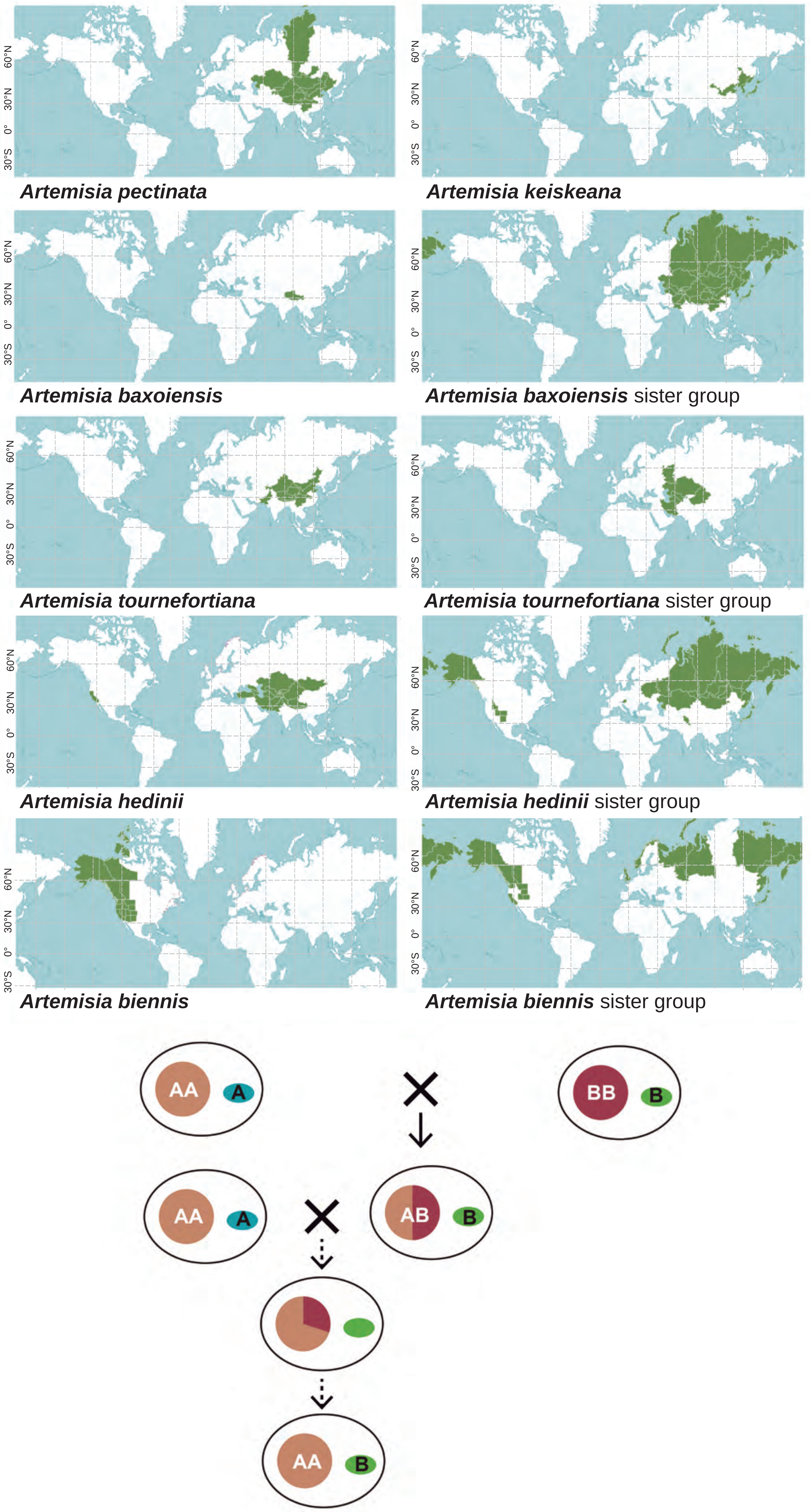

Supplementary Figure 11. Hypothetical scenario for chloroplast capture of *Artemisia* subg. *Pectinatae* species.

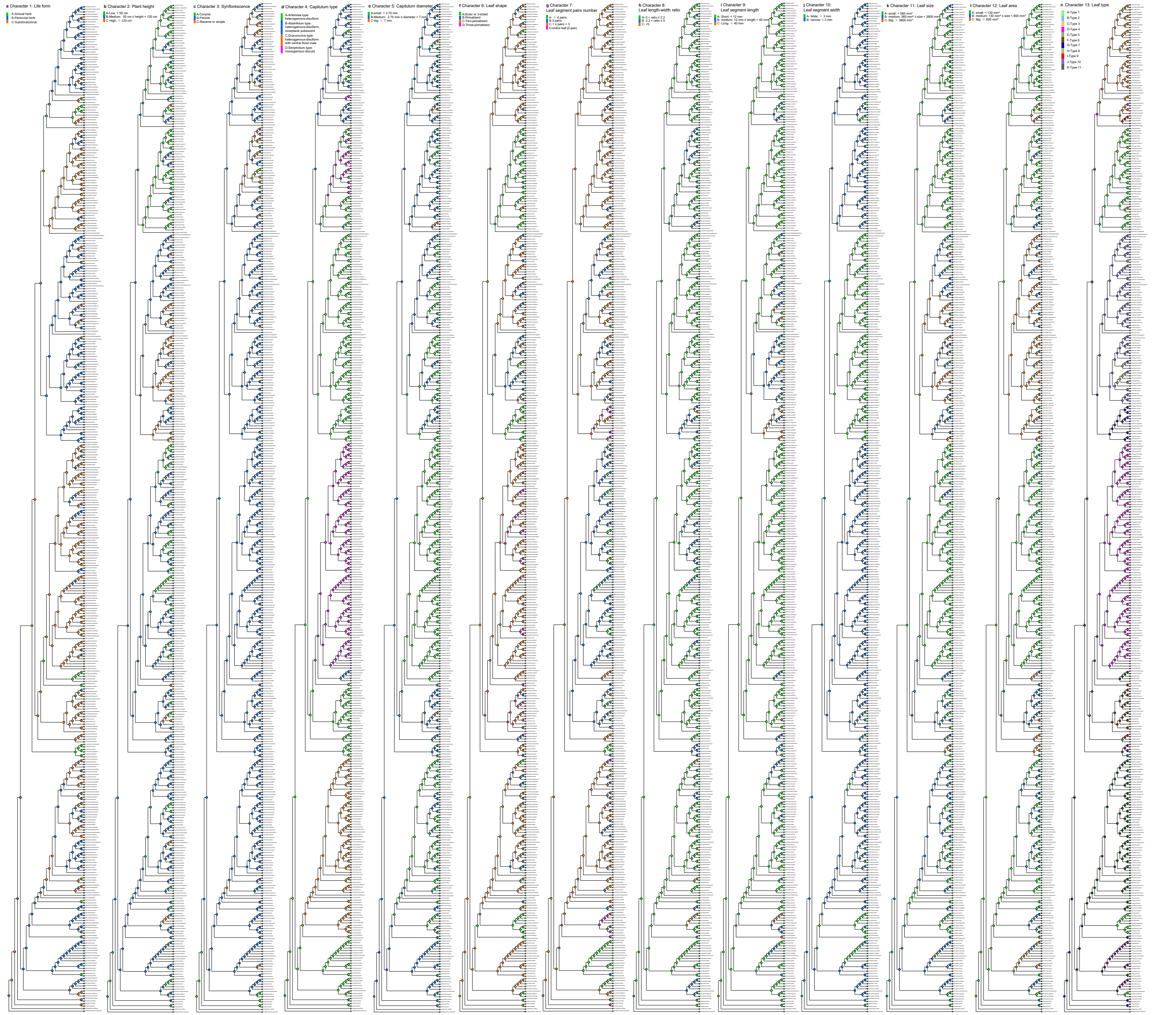

Supplementary Figure 12. Evolutionary trajectories of 13 macromorphological character in *Artemisia* and allied species. Ancestral character states were reconstructed in RASP 3.2[2] using the maximum likelihood method based on the newly inferred GM-derived ML tree (Fig. 2). Pie charts at nodes show character state probabilities.**a** Life form. **b** Plant height. **c** Synflorescence. **d** Capitulum type. **e** Capitulum diameter. **f** Leaf shape. **g** Number of leaf segment pairs. **h** Leaf length-width ratio. **i** Leaf segment length. **j** Leaf segment width. **k** Leaf size. **l** Leaf area. **m** Leaf type. *As RASP requires character states to be represented by uppercase letters, we use uppercase letters rather than numbers to denote character states in this figure.

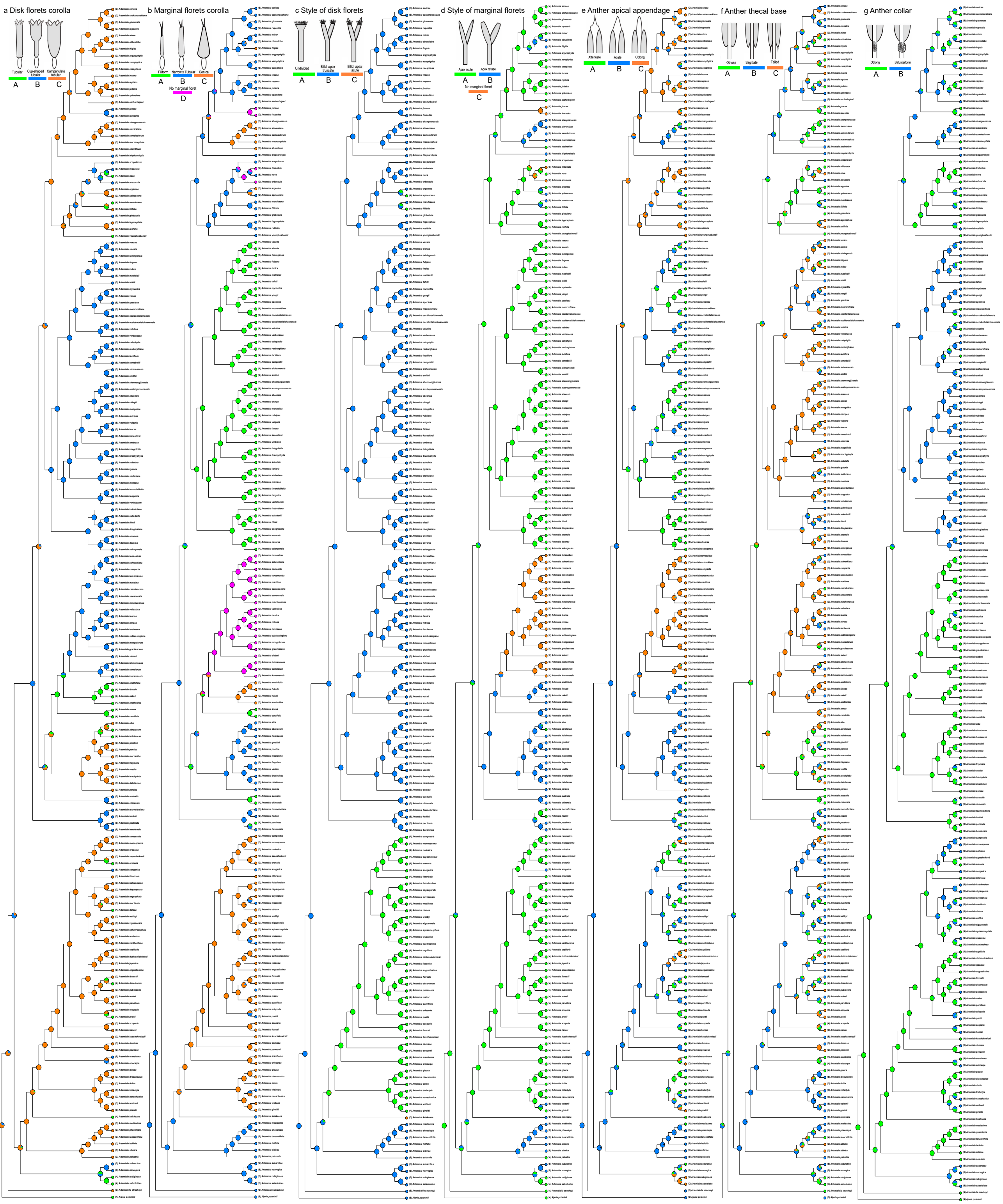

Supplementary Figure 13. Evolutionary trajectories of seven micromorphological characters in *Artemisia* and allied species. Ancestral character states were reconstructed in RASP 3.2[2] using the maximum likelihood method based on the newly inferred GM-derived ML tree (Fig. 2). Pie charts at nodes depict character state probabilities. **a** Corolla shape of disk floret; **b** Corolla shape of marginal floret; **c** Style morphology of disk floret; **d** Style morphology of marginal floret; **e** Shape of anther apical appendage; **f** Shape of anther thecal base; **g** Shape of anther collar.

*As RASP requires character states to be represented by uppercase letters, we use uppercase letters rather than numbers to denote character states in this figure.

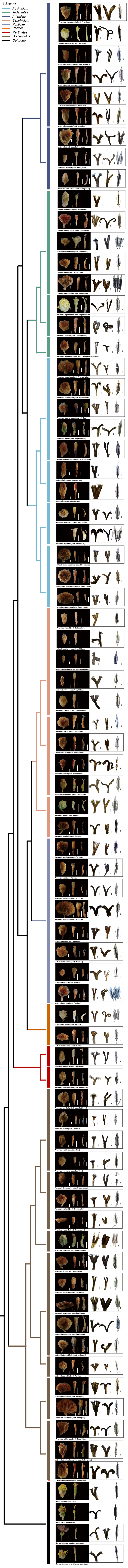

Supplementary Figure 14. Micromorphological characters in *Artemisia* and allied species, depicting morphological variations in capitula, disk florets, marginal florets, style of disk florets, style of marginal florets, and anthers. Simplified sectional phylogenetic relationships are shown based on the GM-derived ML tree in Fig. 2. Scale bar = 1 mm (white) and 100 μm (black). Images represent typical results from three individuals per species, three capitula per individual and three florets per capitulum.

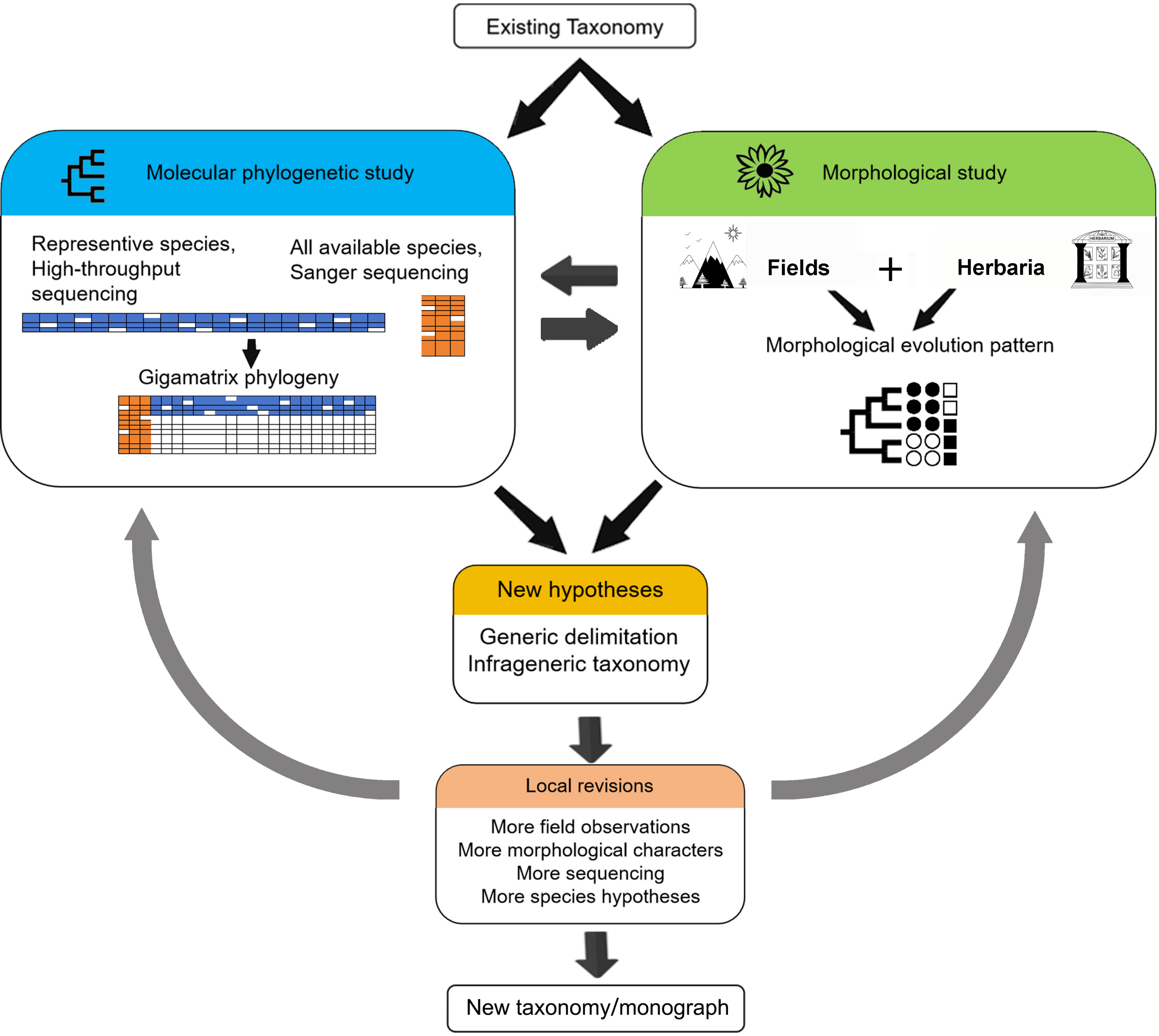

Supplementary Figure 15. Workflow for taxonomic revision of big genera in the genomic era.

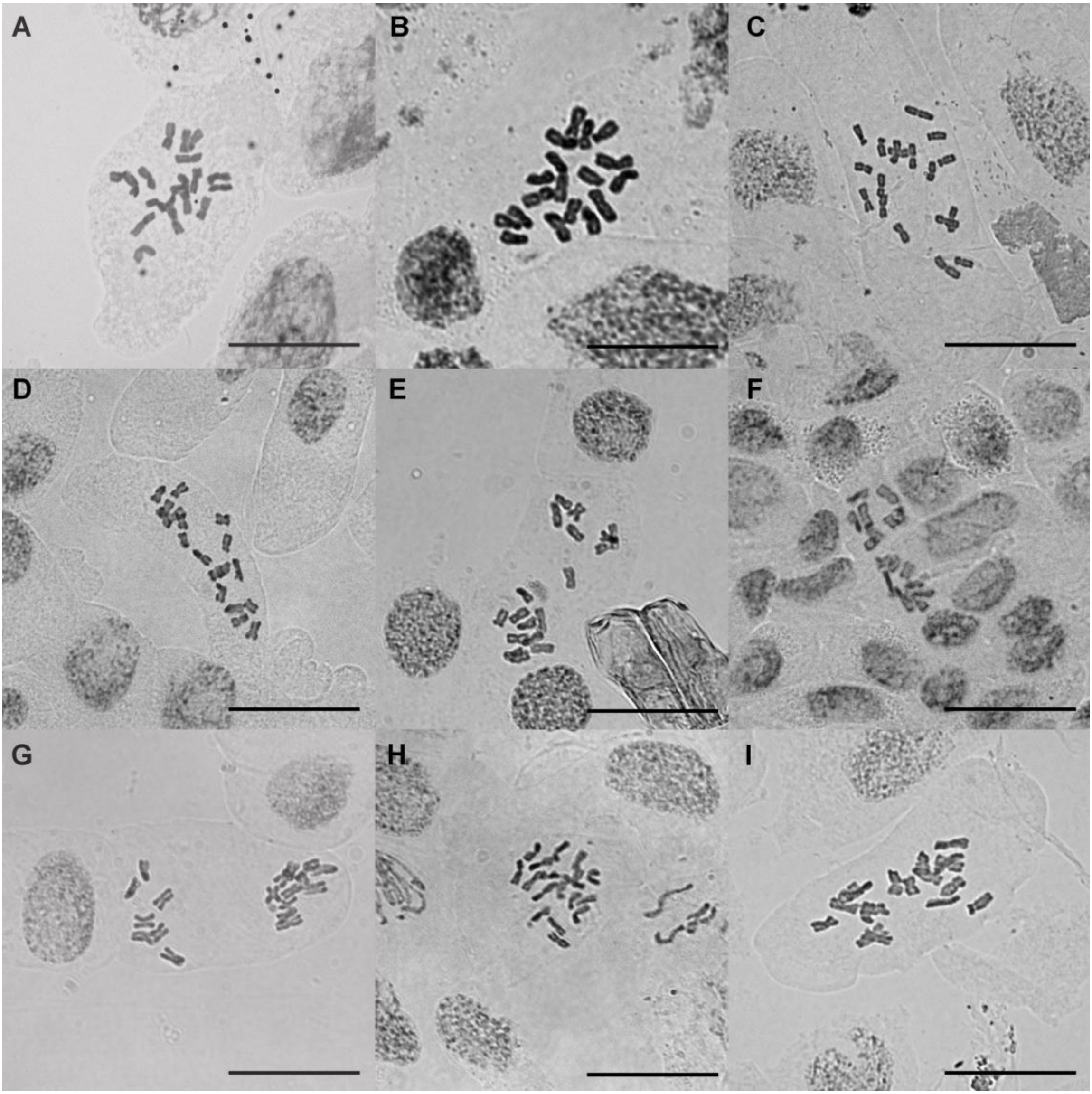

Supplementary Figure 16. Metaphase plates of *Artemisia* and allied species. A. *Artemisia chinensis* (2*n* = 18), B. *Artemisia lagocephala* (2*n* = 18), C. *Artemisia minchunensis* (2*n* = 18), D. *Artemisia sieversiana* (2*n* = 18), E. *Artemisia sphaerocephala* (2*n* = 18), F. *Artemisia stolonifera* (2*n* = 18), G. *Artemisia tournefortiana* (2*n* = 18), H. *Artemisia vestita* (2*n* = 18), I. *Chrysanthemum indicum* (2*n* = 18). Images represent typical results from ≥ 5 root tips per species, with ≥ 3 metaphase plates examined per tip. Scale bar = 20 μm.

Supplementary Table 1. Number of literature records on chemistry of *Artemisia* species (2001–2023). Data derived from Web of Science using search terms "Artemisia" AND "chemistry", retrieved 24 April 2024.

**Year**	**Number of literature records on chemistry of *Artemisia* plants**	**Year**	**Number of literature records on chemistry of *Artemisia* plants**
2001	112	2013	584
2002	137	2014	840
2003	154	2015	1,379
2004	158	2016	1,546
2005	166	2017	1,906
2006	226	2018	1,363
2007	226	2019	1,073
2008	435	2020	969
2009	422	2021	872
2010	359	2022	859
2011	482	2023	764
2012	502		

Supplementary Table 2. Comparison of alternative phylogenetic hypotheses. Three statistical tests and their *p* values generated in IQ-TREE: Shimodaira-Hasegawa (SH) test[3], Kishino-Hasegawa (KH) test[4], and the approximately unbiased (AU) test[5]. All tests were performed as two-tailed tests. The symbol "+" indicates topologies within the 95% confidence set ($P \geq 0.05$); "-" denotes significantly different topologies ($p < 0.05$) that are rejected relative to the ML tree.

**Data**	**Hypothesis**	**Δ log L**	**SH**	**KH**	**AU**
NLC data (low-copy nuclear genes)	*Artemisia* monophyletic	0	1.000+	0.723+	0.760+
	Artemisia + *Artemisiell*a	187.85	0.525+	0.278+	0.388+
	Artemisia + *Artemisiella* + *Ajania* + *Chrysanthemum* + *Elachanthemum*	632.51	0.09+	0.044-	0.053+
plastome data	*Artemisia* monophyletic	362.19	0.000-	0.000-	0.000-
	Artemisia + *Artemisiella*	35.748	0.314+	0.015-	0.000-
	Artemisia + *Artemisiella* + *Ajania* + *Chrysanthemum* + *Elachanthemum*	0	1.000+	0.979+	0.997+

Supplementary Table 3. Eleven leaf types in *Artemisia*.

**Type**	**Name**	**Leaf size**	**Leaf area**	**Leaf shape**	**Leaf length-width ratio**	**Leaf segment pairs number**	**Leaf segment shape**
1	Trilobed small leaves	Small to medium (10–1200 mm²)	Small to medium (10–540 mm²)	3-lobed or entire	> 1	0–1 (2)	leaf/lobe linear-lanceolate, or lanceolate, length 3–10 mm, width 1–3 mm
2	1-pinnatisect multiple-lobed small leaves	Small (30–600 mm²)	Small (10–200 mm²)	1- or 2-pinnatisect	1.0–3.0	2–4	linear, length 3–10 mm, width 1–2 mm
3	2-palmate medium leaves	Small to medium (10–1200 mm²)	Small (10–130 mm²)	2-palmately pinnatisect, the central lobe is often slightly shorter than the lateral lobe	0.7–3.0	2–3	linear, length 2–8 mm, width 1–2 mm
4	2-pinnatisect, multiple-lobed medium long leaves	Small to medium (10–1200 mm²)	Small, rarely medium (10–600 mm²)	2- or 3-pinnatisect	(1)1.5 -3.5(5)	3–4(6)	linear, length 2–5(10) mm, width 0.5–1 mm
5	2-pinnatisect medium leaves	medium, rarely small (100–3000 mm²)	Small to medium (20–500 mm²)	2-pinnatisect	1.0–2.5(10)	3–4(5)	linear or filiform, length 5–20 mm, width 1–2 mm
6	2-pectinately pinnatisect medium leaves	Medium (600–2500 mm²)	Medium to big (150–1300 mm²)	2- or 3- pectinately pinnatisect	1.0–2.0	3–4(5)	toothed lanceolate, length 5–15 mm, width 1.5–3 mm
7	Entrie toothed medium leaves	medium, rarely big (600–6000 mm²)	Medium to big (400–3000 mm²)	3-lobed or entire, margin toothed	(1.5)2–5(20)	0 (1–2)	leaf/lobe lanceolate, length 10–70 mm, width 3–30 mm
8	5-lobed large leaves	medium or big (1000–6000 mm²)	Big, rarely medium (500–2300 mm²)	5-lobed	1.0–2.0	2	lanceolate, length 10–30 mm, width 5–15 mm
9	1-pinnatisect ovate lobe big leaves	medium, rarely big (1500–4000 mm²)	Big (1000–2000 mm²)	1- or 2-pinnatipartite	1.0–2.0	2–3	oblong, length 10–20 mm, width 2–5 mm
10	1-pinnatisect broad-lobed large leaves	medium to big (600–9000 mm²)	Medium to big (300–2000 mm²)	1- or 2-pinnatipartite	1.0–2.0	2–3 (6)	lanceolate or toothed lanceolate, length 10–50 mm, width 3–15 mm
11	2-pectinately pinnatisect large leaves	Medium to big (1500–8000 mm²)	Medium to big (280–2000 mm²)	2-pectinately pinnatisect	1.5–3.5	4–6	toothed lanceolate, , length 6–20 mm, width 2–4 mm

Supplementary Table 4. Macromorphological characters and states of *Artemisia* used in this study.*

No.	Character	Character states
1	Life form	1) Annual herbs; 2) Perennial herbs; 3) Subshrub/shrub
2	Plant height	1) Low: < 50 cm; 2) Medium: 50 cm ≤ height < 120 cm; 3) Height: ≧ 120 cm
3	Capitulum type	1) *Artemisia* type: heterogamous-disciform;<br>2) *Dracunculus* type: heterogamous-disciform with central floret male;<br>3) *Absinthium* type: heterogamous-disciform, receptacle pubescent;<br>4) *Seriphidium* type: homogamous-discoid
4	Synflorescence	1) Corymb; 2) Panicle; 3) Raceme or single
5	Capitulum diameter	1) Small: < 2.75 mm; 2) Middle: 2.75 mm ≤ diameter < 7 mm; 3) Big: ≧ 7 mm
6	Leaf shape	1) Entire/3-lobed; 2) 1-pinnate; 3) 2-pinnate; 4) 3-pinnate
7	Number of leaf segment pairs	1) ≧ 4 pairs; 2) 3 pairs; 3) 1 ≤ pairs < 3; 4) entire leaf (0 pair)
8	Leaf size	1) Small: < 560 mm^2; 2) Medium: 560 mm^2 ≤ size < 3600 mm^2; 3) Big: ≧ 3600 mm^2
9	Leaf area	1) Small: < 130 mm^2; 2) Medium: 130 mm^2 ≤ size < 600 mm^2; 3) Big: ≧ 600 mm^2
10	Leaf length-width ratio	1) 0 < ratio ≤ 2.2; 2) 2.2 < ratio ≤ 5; 3) ratio > 5
11	Leaf segment length	1) Short: < 12 mm; 2) Medium: 12 mm ≤ length < 40 mm; 3) Long: ≧ 40 mm
12	Leaf segment width	1) Wide: ≧ 3 mm; 2) Narrow: < 3 mm
13	Leaf type	1) Type 1: Trilobed small leaf; 2) Type 2: 1-pinnatisect multiple-lobed small leaf; 3) Type 3: 2-palmate medium leaf; 4) Type 4: 2-pinnatisect, multiple-lobed medium long leaf; 5) Type 5: 2-pinnatisect medium leaf; 6) Type 6: 2-pectinately pinnatisect medium leaf; 7) Type 7: Entire toothed medium leaf; 8) Type 8: 5-lobed large leaf; 9) Type 9: 1-pinnatisect ovate lobe big leaf; 10) Type 10: 1-pinnatisect broad-lobed large leaf; 11) Type 11: 2-pectinately pinnatisect large leaf.

*All leaf-related characters were measured from basal stem leaves.

Supplementary Table 5. Micromorphological characters and states of *Artemisia* used in this study.

No.	Character	Character states
1	Corolla shape of disk floret	1) Cup-shaped tubular; 2) Tubular; 3) Campanulate tubular
2	Corolla shape of marginal floret	1) Filiform; 2) Narrow tubular; 3) Conical
3	Style morphology of disk floret	1) Undivided; 2) Bifid, apex truncate; 3) Bifid, apex acute
4	Style morphology of marginal floret	1) Apex acute; 2) Apex retuse
5	Shape of anther apical appendage	1) Attenuate; 2) Acute; 3) Oblonhg
6	Shape of anther thecal base	1) Obtuse; 2) Sagittate; 3) Tailed
7	Shape of anther collar	1) Oblong; 2) Balusterform

Supplementary Method 1

Determine the ploidy level through chromosome counting

We determined the ploidy level of nine diploid species via cytological studies. Plants were first cultivated in the greenhouse of the Institute of Botany, Chinese Academy of Sciences. Fast-growing root tips were then cut, pretreated in a solution with 8-hydroxyquinoline and colchicine in a ratio of 1:1 for 3 hours in the dark, and fixed in Carnoy I (glacial acetic acid:absolute ethanol, 1:3) at 4 °C for 1 h. Next, they were macerated in 1 mol/L hydrochloric acid at 65°C for 8 minutes, and then soaked in clean water for more than 10 minutes, and squashed for chromosome observations. A Leica DM1000 microscope (Leica Microsystems, Wetzlar, Germany) was used for chromosome examination and photography. All diploid species are marked with an asterisk (*) before their names in Supplementary Data 2. Chromosome images of all identified diploid species are included in the Supplementary Materials (Supplementary Figure 16). We also checked the chromosome number of these taxa in the Index to Chromosome Numbers in chromosome counting database (CCDB)[6].

Supplementary Method 2

PCR Reaction and Sequencing

Primers used for PCR amplification were ITS5 and ITS4[7] for the ITS region. Ast-1[8] and 18S-ETS[9] were used to amplify ETS. PCR was performed on a Veriti 96 well thermal cycler (Applied Biosystems, Foster City, California). For ITS amplification, the initial temperature was 95°C for 2 min, then 40 cycles of 96°C for 10 s, 48°C for 30 s, and 72°C for 20 s with a 4-sec extension per cycle. Final extension was at 72°C for 7 min. For ETS, the initial hold at 96°C was for 1 min, followed by the same cycling conditions used for amplifying the ITS region except for an annealing temperature of 60°C. Purification of the PCR products and sequencing were performed by Biomed Co., Beijing.

Supplementary Note 1. An updated infrageneric taxonomy of *Artemisia*

We present an updated infrageneric taxonomy for *Artemisia* integrating morphological and molecular data (Figures 2–5 in the main text), with eight subgenera and 24 sections recognized and keyed out. The complete taxonomy, including all accepted species assigned to each subgenus and section, is provided here. More details for the treatments, including the evidence for species placement are shown in Supplementary Data 6. Species within each section are listed alphabetically.

Artemisia L., Sp. Pl. 2: 845. 1753 – **Type**: *Artemisia vulgaris* L.
= *Crossostephium* Less. in Linnaea 6: 220. 1831 – Type: *Crossostephium chinense* (L.) Makino (≡ *Artemisia chinensis* L.).
= *Picrothamnus* Nutt. in Trans. Amer. Philos. Soc. ser. 2, 7: 417. 1841. – Type: *Picrothamnus desertorum* Nutt. (≡ *Artemisia spinescens* D.C.Eaton).
= *Sphaeromeria* Nutt. in Trans. Amer. Philos. Soc., n.s., 7: 401. 1841. – Type: *Sphaeromeria capitata* Nutt. (≡ *Artemisia capitata* (Nutt.) Sòn.Garcia, Garnatje, McArthur, Pellicer, S.C.Sand).
= *Artemisiastrum* Rydb. in N. Amer. Fl. 34(3): 285. 1916. – Type: *Artemisiastrum palmeri* Rydb. (≡ *Artemisia palmeri* A.Gray).
= *Chamartemisia* Rydb. in N. Amer. Fl. 34(3): 242. 1916. – Type: Chamartemisia compacta Rydb. (≡ *Artemisia constricta* Sòn.Garcia, Garnatje, McArthur, Pellicer, S.C.Sand).
= *Vesicarpa* Rydb. in N. Amer. Fl. 34(3): 242. 1916. – Type: *Vesicarpa potentilloides* (A.Gray) Rydb. (≡ *Artemisia potentilloides* A.Gray).
= *Filifolium* Kitam. in Acta Phytotax. Geobot. 9: 157. 1940 – Type: *Filifolium sibiricum* (L.) Kitam. (≡ *Artemisia sibirica* (L.) Maxim.).
= *Neopallasia* Poljak. in Not. Syst. Herb. Inst. Blot. Acad. Sc. URSS. 17: 429. 1955. – Type: *Neopallasia pectinata* (Pall.) Poljak. (≡ *Artemisia pectinata* Pall.).
= *Kaschgaria* Poljak. in Not. Syst. Herb. Inst. Bot. Ac. Sc. URSS 18: 282. 1957. – Type: *Kaschgaria brachanthemoicles* (C. Winkl.) Poljak. (≡ *Artemisia brachanthemoides* C. Winkl.).
= *Mausolea* Poljakov in Trudy Inst. Bot. Akad. Nauk Kazakhsk. S. S. R. 11: 170. 1961. – Type: *Mausolea eriocarpa* (Bunge) Poljak. (≡ *Artemisia eriocarpa* Bunge).
= *Turaniphytum* Poljakov in Fl. URSS 26: 632, 880. 1961. – Type: *Turaniphytum eranthemum* (Bunge) Poljak. (≡ *Artemisia eranthema* Bunge).
= *Ajaniopsis* Shih in Acta Phytotax. Sin. 16(2): 87. 1978. – Type: *Ajaniopsis penicilliformis* Shih (≡ *Artemisia penicilliformis* (Shih) M. Wei & T. G. Gao).
Unplaced species: *Artemisia avarica* Minatul., *A. dipsacea* Krasch., *A. galinae* Ikonn.
Notes: These three species remain unplaced to any subgenus or section due to insufficient morphological and molecular evidence, primarily resulting from obscure protologues or inaccessibility of any specimens. Further taxonomic research is needed to resolve their placements.

Leaf size
Leaf size was calculated as leaf length × width × 0.75.
Small leaf: < 560 mm²; medium leaf: 560–3600 mm²; big leaf: ≧ 3600 mm².

Key to the subgenera and sections of *Artemisia*

1. Subshrubs, or shrubs; disk florets bisexual and fertile; receptacles glabrous.................................... 2
   2. Achene ribbed; coroniform pappus sometimes present (in *A. chinensis* and *A. kauaiensis*);

distributed in littoral habitats in Southeast Asia and littoral to subalpine habitats in the Hawaiian Islands.. ***Artemisia* subg. *Pacifica* sect. *Pacifica***

2. Achene not ribbed; pappus absent; not distributed in littoral habitats in Southeast Asia or Hawaiian Islands.. 3

3. Leaves pectinately pinnatisect; receptacles glabrous; capitula disciform; disk florets corolla campanulate; marginal florets corolla conical.. .... ***Artemisia* subg. *Ponticae* sect. *Ponticae***

3. Leaves 3-lobed or pinnatisect; receptacles glabrous or rarely pubescent; capitula disciform or discoid; disk florets corolla tubular; marginal florets corolla conical narrow tubular or no marginal floret.. 4

4. Receptacles glabrous; capitula disciform or discoid; New World distribution...................... .... ***Artemisia* subg. *Tridentatae* sect. *Tridentatae***

4. Receptacles pubescent; capitula disciform; old world distribution.................................... 5

5. Leaves pinnatisect; small; capitula diameter < 5 mm.. .... ***Artemisia* subg. *Tridentatae* sect. *Younghusbandianae***

5. Leaves 3-lobed or entire; medium; capitula diameter ≧ 7 mm.................................. .... ***Artemisia* subg. *Tridentatae* sect. *Lagocephalae***

1. Annuals, biennials, perennials; disk florets bisexual and fertile or functionally staminate (not setting fruits); receptacles pubescent or glabrous (if shrub, then disk florets functionally staminate) 6

6. Annuals or biennials; leaves pectinately pinnatisect; capitula sessile; synflorescence panicle........ 7

7. Leaves pinnatisect, with 3 segment pairs, small; blades filiform, linear..................................... .... ***Artemisia* subg. *Pectinatae* sect. *Pectinatae***

7. Leaves 2-pinnatisect, with more than 4 segment pairs, big; blades oblong.................................. .... ***Artemisia* subg. *Pectinatae* sect. *Hedinianae***

6. Perennials, subshrubs, or shrubs, rarely annuals or biennials; leaves entire to pinnatisect; capitula stalked, rarely sessile; synflorescence raceme, panicle or simple... 8

8. Capitula disciform, rarely discoid.. 9

9. Capitula oblate; receptacles glabrous; disk florets bisexual and fertile, corolla funnel-form; marginal florets corolla narrow tubular; leaves entire or pinnatisect, blade linear, width > 3 mm 10

10. Leaves entire, 3-lobed or partite; capitula diameter < 2.75 mm.................................... .... ***Artemisia* subg. *Artemisia* sect. *Selengenses***

10. Leaves pinnatisect; capitula diameter ≧ 2.75 mm... .... ***Artemisia* subg. *Artemisia* sect. *Artemisia***

9. Capitula globose, ovoid, or hemisphere; receptacles pubescent or glabrous; disk florets bisexual and fertile or functionally staminate (not setting fruits), corolla tubular or campanulate; marginal florets corolla tubular or conical, not narrow tubular; leaves entire, pinnatisect, or pectinately pinnatisect; blade linear or filiform, width < 3 mm... 11

11. Receptacles pubescent, rarely glabrous; capitula disciform, rarely discoid; phyllaries almost herbaceous, hairy; disk florets bisexual and fertile; leaves pinnatisect.............. 12

12. Annuals, biennials; capitula disciform or discoid.. 13

13. Capitula discoid.............................. ***Artemisia* subg. *Absinthium* sect. *Juncea***

13. Capitula disciform.. 14

14. Capitula globose, or hemisphere; diam. > 4 mm; receptacles pubescent, rarely

glabrous; leaf length-width ratio ≤ 2.2, leaf segment < 3 pairs................................ ....***Artemisia* subg. *Absinthium* sect. *Sieversianae***

14. Capitula oblate; diam. < 3 mm; receptacles glabrous; leaf length-width ratio > 5, leaf segment ≧ 4 pairs.. ....***Artemisia* subg. *Absinthium* sect. *Blepharolepis***

12. Perennials; capitula disciform...15

15. Plants height > 50 cm; leaves big, leaf segments length ≧ 12 mm, width ≧ 3 mm..***Artemisia* subg. *Absinthium* sect. *Absinthium***

15. Plants height < 50 cm; leaves big, leaf segments length < 12 mm, width < 3 mm......................................***Artemisia* subg. *Absinthium* sect. *Argyrophyllae***

11. Receptacles glabrous; capitula disciform; phyllaries almost coriaceous, glabrous, rarely hairy; disk florets functionally staminate (not setting fruits); leaves entire, pinnatisect, or pectinately pinnatisect..16

16. Plants scattered stellate pubescent; leaves 3-lobed to pinnatisect............................... ....***Artemisia* subg. *Dracunculus* sect. *Salsoloides***

16. Plants not scattered stellate pubescent; leaves entire, or 1–2 pinnatisect................17

17. Annuals, biennials; disk florets bisexual and fertile... ....***Artemisia* subg. *Dracunculus* sect. *Auratae***

17. Perennials, subshrubs, or shrubs; disk florets bisexual and fertile, or functionally staminate (not setting fruits)... 18

18. Perennials; disk florets bisexual and fertile; style of disk floret divergent, ovaries present, not minute... 19

19. Leaves partite; capitula diam. < 4 mm; disk florets corolla tubular; apex of disk florets style acute, not truncate, with triangular appendages........................ ....***Artemisia* subg. *Dracunculus* sect. *Paniculigerae***

19. Leaves pinnatisect, or pectinately pinnatisect; capitula diam. > 4 mm; disk florets corolla campanulate; apex acute of disk florets style truncate, without triangular appendages..20

20. Leaves pinnatisect; segments 2–5 pairs.. .... ***Artemisia* subg. *Dracunculus* sect. *Norvegica***

20. Leaves pectinately pinnatisect; segments more than 6 pairs..................... ....***Artemisia* subg. *Dracunculus* sect. *Laciniatae***

18. Perennials, subshrubs, or shrubs; disk florets functionally staminate (not setting fruits); style of disk floret not divergent, ovaries minute or absent................. 21

21. Leaves entire, or 3–5 lobed; leaf segments lanceolate, width ≧ 3 mm......... ....***Artemisia* subg. *Dracunculus* sect. *Dracunculus***

21. Leaves pinnatisect; leaf segments linear, width < 3 mm............................. ....***Artemisia* subg. *Dracunculus* sect. *Latilobus***

8. Capitula discoid, rarely disciform...22

22. Perennials, or subshrubs; receptacles glabrous; capitula discoid; anther apical appendages oblong; leaves small.. ....***Artemisia* subg. *Seriphidium* sect. *Seriphidium***

22. Annuals, or biennials; receptacles pubescent or glabrous; capitula disciform; anther apical appendages acute; leaves big...23

23. Receptacles pubescent; plants height < 80 cm; leaves 2-pinnatisect..........
....***Artemisia* subg. *Seriphidium* sect. *Anethifoliae***

23. Receptacles glabrous; plants height > 120 cm; leaves 3-pectinately pinnatisect...............................***Artemisia* subg. *Seriphidium* sect. *Annuae***

Synopsis of the subgenera and sections of global *Artemisia* L.

1 ***Artemisia*** subg. ***Dracunculus*** Besser, Bull. Soc. Imp. Naturalistes Moscou. 1: 223. 1829. emend. B. H.Jiao & T.G.Gao, Ann. Bot. 131: 879. 2023. **Type:** *A. dracunculus* L.

Artemisia L. subgenus *Dracunculus* (Bess.) Peterm. Deutschl. Fl. 294. 1848 ≡*Artemisia* sect. *Dracunculus* Bess., Bull. Soc. Nat. Mosc. 1(8): 223. 1829. Type: *Artemisia dracunculus* L. = *Oligosporus* Cass., Bull. Sci. Soc. Philom. Paris. 33. 1817. Type: *Oligosporus campestris* (L.) Cass. (≡ *Artemisia campestris* L.)

Description: Perennials or subshrubs, rarely annuals or biennials; 10–150 cm high; fibrous rooted or taprooted, caudices woody, perennial herbaceous species with rhizomes. Stems erect, rarely procumbent. Leaves deciduous, usually cauline, sometimes basal. Leaf morphology includes types 1, 5, 7, 8, 10, 11. Synflorescence usually panicle, sometimes raceme or corymb. Capitula *Dracunculus* or *Artemisia* type. Achenes without crown and ribs.

Species number and distribution: ca. 122 species; mainly distributed in temperate regions of Eurasia, a few extending to Northern America and Northern Africa.

Notes: *A.* subg. *Dracunculus* was previously characterized by the single key character, i.e. disk florets functionally staminate (not setting fruits). However, the molecular phylogenetic analysis revealed that the previously defined *A.* subg. *Dracunculus* is not monophyletic[10,11]. With the exception of *A. filifolia*, which is embedded within *A.* subg. *Tridentatae*, all the other species of the previous *A.* subg. *Dracunculus* clustered together with some species of *A.* subg. *Artemisia*. They formed the first diverging clade of the genus *Artemisia*. As currently defined, *A.* subg. *Dracunculus* was expanded to include perennials, pectinately pinnatisect large leaves species of *A.* subg. *Artemisia* (e.g. *A. latifolia* and *A. tanacetifolia*), annual, pinnatisect large leaves species of the *A.* subg. *Artemisia* (*A. aurata* and *A. palustris*), *A. keiskeana*, as well as some previously separated genera, such as *Filifolium* and *Kaschgaria*. Most species of *A.* subg. *Dracunculus* grow in semi-arid grassland habitats, a few species in arid deserts (such as *A. eriocarpa* and *A. eranthema*) or in forest (such as *A. angustississima* and *A. japonica*). *Artemisia* subg. *Dracunculus* exhibits a diverse range of morphological characters, rendering any single character—such as life form, leaf shape or size—insufficient to fully encapsulate this clade. Consequently, a combination of morphological characters is required. A comprehensive morphological analysis, though currently absent, is crucial for deep understanding of this big subgenus.

1.1 ***Artemisia*** sect. ***Auratae*** (Poljak.) B.H.Jiao & T.G.Gao, **stat. nov.** [member of subg. *Dracunculus*] ≡ *Artemisia* ser. *Auratae* Poljak., Fl. USSR 26: 491. 1961. **Type**: *A. aurata* Komar.

Description: Annual herbs; 30–70 cm high. Stems single, erect, glabrous. Middle stem leaves type 5: 2- or 3-pinnatisect, segments 2–5 pairs, lobules narrowly linear-lanceolate or filiform. Synflorescence broad panicle. Capitula *Artemisia* type, sessile, usually 2–3 mm in diam.; disk florets bisexual and fertile. Disk florets corolla campanulate tubular; marginal florets corolla narrow tubular; style of disk florets bifid, apex truncate; style of marginal florets apex acute; anther apical appendage acute; anther thecal base sagittate; anther collar oblong.

Species number and distribution: Two species; Northeast China, Far East Russia, Mongolia, Korean Peninsula and Japan.

Notes: *Artemisia* subg. *Dracunculus* sect. *Auratae* is here established by raising the rank of *A.* sect. *Abrotanum* ser. *Auratae* sensu Poljakov[12] and Ling et al.[13], including two species, *A. aurata* and *A. palustris.* Its subgeneric position was transferred from *A.* subg. *Artemisia* to *A.* subg. *Dracunculus* based our phylogenetic analysis (Figure 2). *Artemisia* sect. *Auratae* is characterized by annual or biennial habit, globose capitula, yellow corolla, and large type 5 leaves. *A. palustris* grows in steppes or forest steppes, while *A. aurata* prefers moist rocky hills.

Species included (2): *Artemisia aurata* Kom., *A. palustris* L.

1.2 ***Artemisia*** sect. ***Salsoloides*** T.G. Leonova. Novosti Sist. Vyssh. Rast. 25: 144. 1988. [member of subg. *Dracunculus*]. **Type**: *A. salsoloides* Willd.

Description: Subshrub, 30–70 cm high; with thick, woody root, developing short, woody, strongly branched, perennial sterile shoots, scattered stellate pubescent. Leaves type 1: small, no more than 3 cm long, middle steam leaves 3–5 lobes or entire. Synflorescence raceme or corymb. Capitula *Artemisia* or *Dracunculus* type, sessile, usually 2–3 mm in diam.; disk florets bisexual and fertile (*Artemisia brachanthemoides*, *A. rubiginosa*) or functionally staminate (*A. salsoloides*). Disk florets corolla tubular; marginal florets corolla narrow tubular; style of disk florets bifid, apex truncate; style of marginal florets apex acute; anther apical appendage oblong; anther thecal base obtuse; anther collar balusterform or oblong.

Species number and distribution: 3 species; Central and Eastern European part of Russia, Kazakhstan, North Caucasus, Northwest China (Xinjiang), and western Mongolia.

Notes: *Artemisia* subg. *Dracunculus* sect. *Salsoloides* includes two species of *Kaschgaria* (*Artemisia brachanthemoides* = *Kaschgaria brachanthemoides*, *A. rubiginosa* = *Kaschgaria komarovii*), and *A. salsoloides.* It is characterized by raceme or corymb synflorescence., but the capitula types of its species are different: *A. salsoloides* is *Dracunculus* type, with disk florets functionally staminate, while the two species of *Kaschgaria* are *Artemisia* type. The monophyly of this section and their relationships were also confirmed in a phylogeny based on ITS+ETS[11]. Pellicer et al.[11] noted that species within this section possess a significantly larger genome than other *Artemisia* species. The 2C value of *A. salsoloides* is 11.40 pg[14], while that of *A. brachantemoides* is 14.09 pg[15]. In contrast, the average genome size of most species in the subgenus *Dracunculus* is approximately 6 pg[14].

Species included (3): *Artemisia brachanthemoides* C.Winkl., *A. rubiginosa* B. H. Jiao & T. G. Gao, *A. salsoloides* Willd.

1.3 ***Artemisia*** sect. ***Norvegica*** (Rydb.) B.H.Jiao & T.G.Gao, **stat. nov.** [member of subg. *Dracunculus*] ≡ *Artemisia* ser. *Norvegica* Rydb., North Am. Fl. 34(3): 261. 1916. **Type**: *A. norvegica* Fries.

Description: Short perennial herbs, 20–60 cm high. Stems few or single, 25–40 cm high, erect. Leaves type 10 or 11: medium, pinnatisect or palmatisect. Synflorescence raceme. Capitula *Artemisia* type, hemispherical, 5–12 mm in diam.; disk florets numerous (50–70). Disk florets corolla cup shaped tubular; marginal florets corolla narrow tubular; style of disk florets bifid, apex truncate; style of marginal florets apex retuse; anther apical appendage acute or attenuate; anther thecal base sagittate or obtuse; anther collar balusterform or oblong.

Species number and distribution: seven species; Arctic region.

Notes: All species of *A.* subg. *Dracunculus* sect. *Norvegica* have bisexual and fertile disk florets and glabrous receptacles, and were placed in *A.* subg. *Artemisia*[12,13]. However, they can be distinguished from *A.* subg. *Artemisia* by having large, oblate capitula (diam. > 5 mm vs oblong capitula, diam. < 4 mm in *A.* subg. *Artemisia*), and leaf segment width < 3 mm (vs leaf segment width > 3 mm in *A.* subg. *Artemisia*). Species of sect. *Norvegica* are restricted to the Arctic region, characterized by short herb

(plant height lower than 30(50) cm), and having medium pinnatisect leaves. These characters can be used to distinguish sect. *Norvegica* from sect. *Laciniatae* (temprate region, plant height higher than 50 cm, and having larger leaves).

Species included (6): *Artemisia bejdemaniae* Leonova, *A. eriantha* Ten., *A. norvegica* Fr., *A. punctigera* Krasch. ex Poljakov, *A. sinanensis* Y.Yabe, *A. subarctica* Krasch.

1.4 ***Artemisia*** sect. ***Laciniatae*** (Kitam.) B.H.Jiao & T.G.Gao, **stat. nov.** [member of subg. *Dracunculus*] ≡ *Artemisia* ser. *Laciniatae* Kitam. in Act. Phytotax. Geobot. 8: 65. 1939. **Type**: *A. laciniata* Willd. (= *A. tanacetifolia* L.)

Description: Perennial herbs; 50–70 cm high; rootstock horizontally creeping to obliquely rising. Leaves Type 11: Basal leaves long petiolate; middle stem leaves petiole 3–12 mm; 2-pectinately pinnatisect; segments 6–8 pairs; lobules toothed lanceolate; sometimes type 5. Synflorescence raceme, narrowly panicle or corymb (e.g. *A. sibirica* = *Filifolium sibiricum*). Capitula *Artemisia* type, hemispherical or oblate, 4–8 mm in diam. Disk florets corolla campanulate tubular; marginal florets corolla narrow tubular; style of disk florets bifid, apex truncate; style of marginal florets apex retuse; anther apical appendage acute or attenuate; anther thecal base sagittate, tailed or obtuse; anther collar oblong or balusterform.

Species number and distribution: 17 species; Siberia and the Far East of Russia, the Korean Peninsula, Japan, and northern China.

Notes: *Artemisia* subg. *Dracunculus* sect. *Laciniatae* has the same capitula characters with *A.* subg. *Dracunculus* sect. *Norvegica*, thus, species of this section were once placed in *A.* subg. *Artemisia*. They can be distinguished from *A.* subg. *Artemisia* by narrowly paniculate or raceme inflorescence (vs broadly paniculate inflorescence), and 2- pectinately pinnatisect leaves (vs 1–2 pinnatisect leaves). Among the species in this section, the morphology of *A. sibirica* (= *Filifolium sibiricum*) is relatively unique by having corymb synflorescence (vs raceme or narrowly panicle) and Type 5 leaves (vs Type 11). However, all molecular phylogenetic analyses conducted so far[1,11] resolved *A. sibirica* as a member of *Artemisia* subg. *Dracunculus* sect. *Laciniatae*.

Species included (17): *Artemisia arctisibirica* Korobkov, *A. longipetiolata* Charit., *A. macrorhiza* Turcz., *A. remotiloba* Krasch. ex Poljakov, *A. transbaicalensis* Leonova, *A. armeniaca* Lam., *A. atrata* Lam., *A. insipida* Vill., *A. laciniata* Willd., *A. latifolia* Ledeb., *A. maximovicziana* Krasch. ex Poljakov, *A. medioxima* Krasch. ex Poljakov, *A. oelandica* (Besser) Krasch., *A. pancicii* Ronniger ex Danihelka & Marhold, *A. phaeolepis* Krasch., *A. sibirica* (L.) Maxim., *A. tanacetifolia* L.

1.5 ***Artemisia*** sect. ***Paniculigerae*** (Poljak.) B.H.Jiao & T.G.Gao, **stat. nov.** [member of subg. *Dracunculus*] ≡ *Artemisia* ser. *Paniculigerae* Poljak. Fl. USSR 26: 487. 1961. **Type**: *A. keiskeana* Miq.

Description: Perennial, 50–100 cm high. Rhizome strong, with underground shoots. Leaves Type 7: leaf blade obovate or broadly cuneate, base attenuate, margin acutely serrate from middle to apex, apex rounded; green above, usually glabrous, pale green beneath, weakly hairy. Synflorescence panicle. Capitula *Artemisia* type, subglobose, 3–3.5 mm in diam.; style arms of disk florets not truncate, with triangular appendages. Disk florets corolla tubular; marginal florets corolla narrow tubular; style of disk florets bifid, apex acute; style of marginal florets apex retuse; anther apical appendage attenuate; anther thecal base sagittate; anther collar oblong.

Species number and distribution: Two species; Northeast China, Eastern Russia, Korean Peninsula, and Japan.

Notes: The species of *A.* subg. *Dracunculus* sect. *Paniculigerae* has *Artemisia* type capitula, and type 7 leaves. It is distinguished from *A.* subg. *Artemisia* by its globose capitula (vs oblong in *A.* subg.

Artemisia). Besides, the apex of the style arm of disk florets of *A. keiskeana* is triangular, not truncate as that in all other species of tribe Anthemideae. The floral micromorphology of *A. pedunculosa* has not been observed.

Species included (1): *Artemisia keiskeana* Miq., *A. pedunculosa* Miq.

1.6 ***Artemisia*** sect. ***Dracunculus***. [member of subg. *Dracunculus*] **Type:** *A. dracunculus* L.

Description: Perennials, subshrubs or shrubs; 30–150 cm high. Perennial species with branched, woody rhizome. Leaves usually type 1 (small, entire or 3-5 lobed); sometimes type 8 (large, 5-lobed to pinnatipartite, *Artemisia dubia*). Synflorescence panicle. Capitula *Dracunculus* type, globose, 3–3.5 mm in diam. Disk florets corolla campanulate tubular; marginal florets corolla conical; style of disk florets undivided; style of marginal florets apex acute; anther apical appendage acute or attenuate; anther thecal base sagittate, tailed or obtuse; anther collar oblong or balusterform.

Species number and distribution: 11 species; temperate regions of Eurasia and North America. *Artemisia dracunculus* and *A. dubia* are widely distributed, while the rest narrowly distributed.

Notes: *Artemisia* subg. *Dracunculus* sect. *Dracunculus*, along with *A.* subg. *Dracunculus* sect. *Latilobus*, forms the core of *A.* subg. *Dracunculus*. The species within these two sections are distinguished by their *Dracunculus* type capitula. Most members of sect. *Dracunculus* are perennial, with only two subshrub species as exceptions. These perennial herbs all possess branched, woody rhizomes. In contrast, all members of sect. *Latilobus* are shrubs. Furthermore, the leaf segment width in sect. *Dracunculus* exceeds 2 mm, while it is less than 1.5 mm in sect. *Latilobus*.

Species included (11): *Artemisia daghestanica* Krasch. & Poretzky, *A. dimoana* Popov, *A. dracunculiformis* Krasch., *A. dracunculus* L., *A. dubia* Wall. ex Besser, *A. giraldii* Pamp., *A. glauca* Pall. ex Willd., *A. kotuchovii* Kupr., *A. nanschanica* Krasch., *A. tridactyla* Hand.-Mazz., *A. waltonii* J.R.Drumm. ex Pamp.

1.7 ***Artemisia*** sect. ***Latilobus*** Y. R. Ling. Act. Phytotax. Sin. 18(4): 512. 1980 [member of subg. *Dracunculus*] **Type:** *A. japonica* Thunb.

Description: Annuals, biennials, perennials, subshrubs or shrubs; 10–150 cm high. Perennial species with branched, woody rhizome. Leaves mostly type 5 (2-pinnatisect medium leaves, with lobes linear or filiform). Synflorescence panicle. Capitula *Dracunculus* type, globose, 1.5–3 mm in diam. Disk florets corolla campanulate tubular; marginal florets corolla conical; style of disk florets undivided; style of marginal florets apex acute; anther apical appendage acute or attenuate; anther thecal base sagittate, tailed or obtuse; anther collar oblong or balusterform.

Species number and distribution: 81 species; temperate regions of Eurasia and North America.

Notes: This expanded *A.* subg. *Dracunculus* sect. *Latilobus* is morphologically most diverse in subg. *Dracunculus*. Besides *Dracunculus* type capitula, the species of this group are characterized by middle stem leaf segments linear or filiform. Most species within this section lack a rhizome.

Species included (81): *Artemisia abolinii* Lazkov, *A. albicerata* Krasch., *A. borealis* Pall., *A. chienshanica* Ling & W.Wang, *A. congesta* Kitam., *A. gyangzeensis* Ling & Y.R.Ling, *A. hallaisanensis* Nakai, *A. henriettae* Krasch., *A. karavajevii* Leonova, *A. lipskyi* Poljakov, *A. nortonii* Pamp., *A. olgensis* (Vorosch) Vorosch., *A. oligocarpa* Hayata, *A. pannosa* Krasch., *A. pengchuoensis* Y.R.Ling & S.Y.Zhao, *A. pewzowii* Krasch., *A. quinqueloba* Trautv., *A. remosa* Sugaw., *A. richardsoniana* Besser, *A. stricta* Edgew., *A. swatensis* Podlech, *A. trautvetteriana* Besser, *A. tschernieviana* Bess., *A. angustissima* Nakai, *A. arenaria* DC., *A. bargusinensis* Spreng., *A. campestris* L., *A. cashemirica* M.K.Kaul & S.K.Bakshi, *A. capillaris* Thunb., *Artemisia crithmifolia* L.,*A. demissa* Krasch., *A. depauperata* Krasch., *A. desertorum* Spreng., *A. dolosa* Krasch., *A. duthreuil-de-rhinsi* Krasch., *A.*

eranthema Bunge, *A. eriocarpa* Bunge, *A. eriopoda* Bunge, *A. flahaultii* Emb. & Maire, *A. forrestii* W.W.Sm., *A. gansuensis* Ling & Y.R.Ling, *A. globosa* Krasch., *A. globosoides* Ling & Y.R.Ling, *A. halodendron* Turcz. ex Besser, *A. hancei* (Pamp.) Ling & Y.R.Ling, *A. implicata* T.G.Leonova, *A. insularis* Kitam., *A. japonica* Thunb., *A. jordanica* Danin, *A. kelleri* Krasch., *A. klementzae* Krasch., *A. kuschakewiczii* C.Winkl., *A. ledebouriana* Besser, *A. limosa* Koidz., *A. littoricola* Kitam., *A. macilenta* (Maxim.) Krasch., *A. mairei* H.Lév., *A. manshurica* (Kom.) Kom., *A. marschalliana* Spreng., *A. monosperma* Delile, *A. morrisonensis* Hayata, *A. mustangensis* Yonek., *A. niitakayamensis* Hayata, *A. ordosica* Krasch., *A. oxycephala* Kitag., *A. parviflora* Roxb. ex D.Don, *A. pewzowi* C.Winkl., *A. prattii* (Pamp.) Ling & Y.R.Ling, *A. pubescens* Ledeb., *A. pycnocephala* DC., *A. pycnorrhiza* Ledeb., *A. saposhnikovii* Krasch. ex Poljakov, *A. scoparia* Waldst. & Kit., *A. songarica* Schrenk, *A. sphaerocephala* Krasch., *A. tomentella* Trautv., *A. tsugitakaensis* (Kitam.) Ling & Y.R.Ling, *A. wellbyi* Hemsl. & H.Pearson, *A. wudanica* Liou & W.Wang, *A. xanthochroa* Krasch., *A. xigazeensis* Y.R.Ling & M.G.Gilbert, *A. yamadae* (Kitam.) Hideki Takah. & Barkalov.

2 ***Artemisia*** subg. ***Pectinatae*** B. H. Jiao & T. G. Gao, Ann. Bot. 131: 879. 2023. **Type**: *A. pectinata* Pall.

Description: Annual or biennial herbs; 12–40 cm or more than 100 cm high. Root vertical. Stems most often few or solitary, erect. Leaves type 2 (small, pinnatisect multiple-lobed in sect. *Pectinatae*) or type 11 (medium to large, pectinately pinnatisect in sect. *Hedinianae*). Synflorescence of short axillary spikes grouped into a slender, leafy panicle. Capitula *Artemisia* type, glabrous. Achenes without crown and ribs.

Species number and distribution: *Artemisia* subg. *Pectinatae* comprises about ten species classified into two sections, and is distributed in temperate regions of Europe, Asia, Africa, North America, and South America. Most species grow in semi-arid grassland at low to medium altitudes, a few species such as *A. hedinii* and *A. baxoinensis* mainly grow in Qinghai-Tibet Plateau.

Notes: *Artemisia* subg. *Pectinatae* is characterized by annual or biennial herbs, pectinately pinnatisect leaves, and capitula in dense narrow panicles. They were separated from the subgenus *Artemisia* by Jiao et al.[1]. However, their positions within the phylogenies based on nuclear genes and plastid genes differ. This discrepancy may be attributed to chloroplast capture. The capitula of this subgenus are densely clustered in spikes on the small branches of the panicles, demonstrating a significant fruiting capacity. For instance, the notorious weed distributed in northwest North America, *Artemisia biennis*, is reported to produce between 400,000 and 1 million achenes per plant annually (Mahoney and Kegode, 2017). This high fruiting capacity could potentially explain why this annual subgenus is distributed across multiple continents. *Artemisia abyssinica* from Arabian Peninsula, *A. schimperi* and *A. tilhoana* from North and Central Africa and *A. klotzschiana* from Mexico were not sampled in our molecular phylogenetic study. We assign them to *Artemisia* subg. *Pectinatae* as they fit well to it morphologically. We acknowledge that further phylogenetic work is required to test the present treatment.

2.1 ***Artemisia*** sect. ***Pectinatae*** [member of subg. *Pectinatae*] **Type**: *A. pectinata* Pall.

Description: Annual or biennial herbs; 12–40 cm high. Root vertical. Stems most often few or solitary, erect. Leaves type 2 (small, pinnatisect multiple-lobed in sect. *Pectinatae*). Synflorescence of short axillary spikes grouped into a slender, leafy panicle. Capitula *Artemisia* type, globose or ovoid. Disk florets corolla cup shaped tubular; marginal florets corolla filiform or narrow tubular; style of disk florets bifid, apex truncate; style of marginal florets apex retuse; anther apical appendage acute or attenuate; anther thecal base sagittate or obtuse; anther collar oblong.

Species number and sistribution: two species; Northwest China, Mongolia, Central Asia, and East

Siberia.

Notes: The two sections of *A.* subg. *Pectinatae* were differentiated by the leaf length: leaf length < 2 cm in *A.* subg. *Pectinatae* sect. *Pectinatae* vs leaf length > 5 cm in *A.* subg. *Pectinatae* sect. *Hedinianae*.

Species included (6): *Artemisia abyssinica* Sch.Bip. ex Oliv. & Hiern, *A. baxoiensis* B.H.Jiao & T.G.Gao, *A. klotzschiana* Besser, *A. pectinata* Pall, *A. schimperi* Sch.Bip. ex Engl., *A. tilhoana* Quézel.

2.2 ***Artemisia*** sect. ***Hedinianae*** (Y. R. Ling) B.H.Jiao & T.G.Gao, **stat. nov.** [member of subg. *Pectinatae*] ≡ *Artemisia* ser. *Hedinianae* Y. R. Ling, Bull. Bot. Res., 8 (4): 17. 1988. **Type**: *Artemisia hedinii* Ostenf.

Description: Annual or biennial herbs; more than 100 cm high or sometime 15–40 cm (*A. hedinii*). Root vertical. Stems most often few or solitary, erect. Leaves Type 11 (medium to large, pectinately pinnatisect). Synflorescence of short axillary spikes grouped into a slender, leafy panicle. Capitula *Artemisia* type, globose. Disk florets corolla cup shaped tubular; marginal florets corolla narrow tubular; style of disk florets bifid, apex truncate; style of marginal florets apex acute; anther apical appendage acute or attenuate; anther thecal base sagittate; anther collar oblong.

Species included (4): *Artemisia biennis* Willd., *A. hedinii* Ostenf., *A. magellanica* Sch.Bip., *A. tournefortiana* Rchb.

Species number and distribution: four species; Central Asia, North America, and South America.

Notes: *Artemisia* subg. *Pectinatae* sect. *Hedinianae* is newly established by raising the rank of the *A.* subg. *Artemisia* sect. *Abrotanum* ser. *Hedinianae* described in *Flora of China*[13]. This section was updated with the inclusion of *A. tournefortiana*, *A. biennis* and *A. magellanica* based on phylogenetic analysis[1].

3 ***Artemisia*** subg. ***Pacifica*** C.R.Hobbs & B.G.Baldwin, J. Biogeogr. 40: 451. 2013. **Type**: *A. australis* Less.

Description: Small shrubs, branches sometimes trailing; 50–100 cm high. Leaves type 9, medium to big, pinnatisect lobe ovate; leaves clustered near tips. Synflorescence panicle or raceme. Capitula *Artemisia* type, globose. Achene conspicuously 5-ribbed, glandular; pappus sometimes present (in *A. chinensis* and *A. kauaiensis*), teeth irregular. Disk florets corolla cup shaped tubular; marginal florets corolla filiform; style of disk florets bifid, apex truncate; style of marginal florets apex retuse; anther apical appendage acute; anther thecal base obtuse; anther collar oblong or balusterform.

Species number and distribution: four species currently known; one species (*A. chinensis*) of littoral habitats in Southeast Asia and three species (*A. australis*, *A. kauaiensis*, and *A. mauiensis*) of littoral to subalpine habitats in the Hawaiian Islands.

Notes: We recognize *Artemisia* subg. *Pacifica* following the circumscription presented by Hobbs and Baldwin[16] without addition or exclusion. It is characterized by shrub habit, leaves clustered near tips, and achene conspicuously 5-ribbed. *A. chinensis* (= *Crossostephium chinense*) was once treated as an independent genus *Crossostephium*. The molecular phylogenetic analysis[16] revealed that *A. chinensis* and 3 other species endemic to the Hawaiian Islands formed a well supported clade.

3.1 ***Artemisia*** sect. ***Pacifica***. [member of subg. *Pacifica*] **Type**: *A. australis* Less.

The range of this section is equivalent to *A.* subg. *Pacifica*.

Species included (4): *Artemisia australis* Less., *A. chinensis* L., *A. kauaiensis* (Skottsb.) Skottsb., *A. mauiensis* Skottsb.

4 ***Artemisia*** subg. ***Ponticae*** B.H.Jiao & T.G.Gao, Ann. Bot. 131: 879. 2023. **Type**: *A. pontica* L.

Description: Subshrubs or shrubs, 60–150 cm high; usually strongly aromatic. Root vertical. Stems

numerous, erect. Leaves type 5 (2-pinnatisect medium leaves, with lobes linear or filiform) or type 6 (2-pectinately medium pinnatisect, with lobes lanceolate). Synflorescence panicle. Capitula Artemisia type, disciform. Achenes without crown and ribs. Disk florets corolla campanulate tubular; marginal florets corolla narrow tubular; style of disk florets bifid, apex truncate; style of marginal florets apex retuse; anther apical appendage acute or oblong; anther thecal base sagittate, tailed or obtuse; anther collar oblong or balusterform.

Species number and distribution: Twenty-three species; widely distributed in temperate regions of Eurasia, with a few species spreading to Africa (e.g. *Artemisia afra*); growing mainly in semi-arid rocky hillsides, with a few species (e.g. *A. molinieri*) in wetlands.

Notes: Members of this recently established subgenus were transferred from *Artemisia* sect. *Abrotanum* referring to Ling et al.[13]. Molecular phylogenetic analysis[16,17] revealed that sect. *Abrotanum* was obviously polyphyletic. Our phylogenomic analysis (Figure 2) showed the shrub species of *Artemisia* sect. *Abrotanum* formed one clade, namely the recently established subgenus *Ponticae*; while the perennial herbaceous species (e.g. *A. tanacetifolia*) were placed in subgenus *Dracunculus*; and the annual herbaceous species were classified into the subgenus *Pectinatae* (e.g. *A. biennis*) and the subgenus *Seriphidium* (*A. annua* and *A. caruifolia*), respectively. Similar to the *A.* subg. *Pectinatae*, there are significant differences in the phylogenetic topologies composed of nuclear and chloroplast data (Figure 3). The chloroplast phylogeny showed the species of *A.* subg. *Ponticae* formed two clades, corresponding exactly to the two subclades of the *A.* subg. *Ponticae* revealed in the nuclear phylogeny (Figures 2 and 3). The geographical distribution of these two subclades is different, with one mainly in Europe and the other mainly in East Asia. We hypothesize that this cytonuclear discordance may be attributable to hybridization. However, we were unable to identify a suite of characters to delineate the two subclades of *A.* subg. *Ponticae*. Given these findings, we believe it is premature to further classify it into two sections. More comprehensive morphological studies on this subgenus are required.

4.1 ***Artemisia*** sect. ***Ponticae*** [member of subg. ***Ponticae***] **Type**: *A. pontica* L.

Morphology, species number and distribution of *Artemisia* sect. *Ponticae* is the same as *Artemisia* subg. *Ponticae* presented above.

Species included (23): *Artemisia abrotanum* L., *A. adamsii* Besser, *A. andersiana* Podlech, *A. afra* Jacq. ex Willd., *A. aksaiensis* Y.R.Ling, *A. alba* Turra, *A. baimaensis* Y.R.Ling & Z.C.Chuo, *A. brachyloba* Franch., *A. chamaemelifolia* Vill., *A. dalai-lamae* Krasch., *A. freyniana* (Pamp.) Krasch., *A. gmelinii* Weber ex Stechm., *A. hololeuca* M.Bieb. ex Besser, *A. macrantha* Ledeb., *A. mesatlantica* Maire, *A. molinieri* Quézel, Barbero & R.J.Loisel, *A. negrei* A.Ouyahya, *A. olchonensis* Leonova, *A. persica* Boiss., *A. polybotryoidea* Y.R.Ling, *A. pontica* L., *A. stechmanniana* Besser, *A. vestita* Wall. ex Besser.

5 ***Artemisia*** subg. ***Seriphidium*** Besser ex Less., Syn. Gen. Compos. 264. 1832. emend. B.H.Jiao & T.G.Gao, Ann. Bot. 131: 879. 2023. **Type**: *A. maritima* L.

Description: Annuals, biennials, subshrubs or shrubs, 30–150 cm high; strongly aromatic. Root vertical. Annuals or biennials stems few (sect. *Anethifoliae*) or single (sect. *Annuae*); subshrubs or shrubs stem numerous (sect. *Seriphidium*), erect. Leaves type 4 (small to medium, 2-pinnatisect, multiple-lobed, sect. *Seriphidium*) type 5 (2-pinnatisect medium leaves, with lobes linear or filiform, sect. *Anethifoliae*), type 6 (2-pectinately medium pinnatisect, with lobes lanceolate, sect. *Annuae*). Synflorescence panicle. Capitula *Artemisia*, *Absinthium* or *Seriphidium* type: disciform (sect. *Anethifoliae, Annuae*) or discoid (sect. *Seriphidium*); receptacles glabrous (sect. *Seriphidium, Annuae*) or pubescent (sect. *Anethifoliae*); disk florets bisexual and fertile. Achenes without crown and ribs.

Species number and distribution: ca. 128 species, Eurasia.

Notes: The present recircumscribed *A.* subg. *Seriphidium* is expanded and composed of three subclades with significant morphological differences. The first is the largest and roughly equal to the original *A.* subg. *Seriphidium*[10], characterized by *Seriphidium* type capitula (discoid capitula without female florets). We treated them as *A.* subg. *Seriphidium* sect. *Seriphidium.* They are the core of *A.* subg. *Seriphidium* with ca. 119 species. The second is the newly raised *A.* subg. *Seriphidium* sect. *Anethifoliae* characterized by annual or biennial habit, *Absinthium* type capitula. The third is the newly established *A.* subg. *Seriphidium* sect. *Annuae*, characterized by *Artemisia* type capitula and large 2–3 pectinately pinnatisect leaves (type 6). *Artemisia* subg. *Seriphidium* is currently the most diverse morphologically within the genus *Artemisia*, with its distribution majoritarily centered in the arid Central Asia region. Most species are narrowly endemic. A taxonomic revision of this complex group is long overdue.

5.1 ***Artemisia*** sect. ***Annuae*** Rydb. N. Amer. Fl. 34 (3): 247. 1916. [member of subg. *Seriphidium*] **Type**: *A. annua* L.

Description: Annuals or biennials; 70–160 (200) cm high, usually strongly aromatic. Root vertical. Stems usually single, erect. Leaves type 6 (medium, 2-pectinately pinnatisect, with lobes lanceolate). Synflorescence panicle. Capitula *Artemisia* type; corolla dark yellow. Disk florets corolla tubular; marginal florets corolla filiform; style of disk florets bifid, apex truncate; style of marginal florets apex retuse; anther apical appendage acute; anther thecal base tailed; anther collar oblong.

Species number and distribution: three species; *Artemisia annua* in Europe and Asia, *A. caruifolia* and *A. calcicola* in East Asia (Southeast China, North India, Japan, Korea, Myanmar, Nepal, North Vietnam).

Notes: *Artemisia* subg. *Seriphidium* sect. *Annuae* is the sister lineage to the rest of subg. *Seriphidium* (Figure 2). The species of this section were placed originally in *A.* subg. *Artemisia* sensu Ling et al.[13] on account of their *Artemisia* type capitula. It is distinguished from *A.* subg. *Artemisia* by its annual or biennial life form, Type 6 leaves, and globose capitula (vs perennial life form, Type 7 or 10 leaves, and oblong capitula in *A.* subg. *Artemisia*).

Species included (3): *Artemisia annua* L., *A. caruifolia* Buch.-Ham. ex Roxb., *A. calcicola* X.Q.Guo & L.Wang.

5.2 ***Artemisia*** sect. ***Anethifoliae*** (Poljak.) B.H.Jiao & T.G.Gao, **stat. nov.** [member of subg. *Seriphidium*] ≡ *Artemisia* ser. *Anethifoliae* Poljak. Fl. USSR 26: 522. 1961. **Type**: *A. anethifolia* Web. ex Stechm

Description: Annuals or biennials; 20–90 cm high, strongly aromatic. Root vertical. Stems few, erect. Leaves type 5 (medium, 2-pinnatisect medium leaves, with lobes linear or filiform). Synflorescence panicle. Capitula *Absinthium* type, obovoid, nodding; corolla dark yellow. Disk florets corolla tubular; marginal florets corolla conical; style of disk florets bifid, apex truncate; style of marginal florets apex retuse; anther apical appendage acute; anther thecal base sagittate or tailed; anther collar oblong.

Species number and distribution: six species; Northeast Asia; growing mainly in the saline-alkali soil of coastal areas or wastelands.

Notes: *Artemisia* subg. *Seriphidium* sect. *Anethifoliae* is characterized by medium leaves, with length > 5 cm; leaf segments linear, longer than 5 mm. Most species of this section have pubescent receptacles. The circumscription of this newly established section is roughly equal to *A.* subg. *Artemisia* sect. *Absinthium* ser. *Anethifoliae* and *A.* subg. *Artemisia* sect. *Abrotanum* ser. *Fukudo* sensu Ling et al.[13].

Species included (6): *Artemisia anethifolia* Weber ex Stechm., *A. anethoides* Mattf., *A. fauriei* Nakai,

A. fukudo Makino, *A. nakaii* Pamp., *A. przewalskii* Krasch.

5.3 ***Artemisia*** sect. ***Seriphidium*** [member of subg. *Seriphidium*] **Type**: *A. maritima* L.

Description: Perennials, subshrubs or shrubs; 30–60 cm high; strongly aromatic. Root vertical, with numerous short fertile branches. Stem numerous, erect. Leaves type 4 (small to medium, 2-pinnatisect, multiple-lobed). Synflorescence panicle or raceme. Capitula *Seriphidium* type, oblong. Disk florets corolla cup shaped tubular; marginal florets corolla absent; style of disk florets bifid, apex truncate; style of marginal florets absent; anther apical appendage oblong; anther thecal base sagittate or tailed; anther collar oblong.

Species number and distribution: ca. 119 species; Eurasia and North Africa; mostly growing in arid areas.

Notes: The circumscription of *Artemisia* subg. *Seriphidium* sect. *Seriphidium* is roughly equal to *A.* subg. *Seriphidium*[10] with the exclusion of three annual species (i.e. *Artemisia deserti*, *A. juncea* and *A. leucodes*), which were treated as a section of *A.* subg. *Absinthium* based on our phylogenetic analysis (Figure 2). Upon examination of the specimens, it was observed that the florets and leaves of sect. *Seriphidium* were not concurrently present, with the leaves almost entirely shedding during the flowering and fruiting phase. This character is likely an adaptation to drought habitat, but it presents certain challenges for morphological observation. The sect. *Seriphidium* represents the most drought-resistant group within the genus *Artemisia*. During the process of diversification, different species may have developed distinct ecological preferences. For instance, *A. camelorum* and *A. pauciflora* are halophytes, while *A. santolina* is a psammophyte[18].

Species included (119): *Artemisia aflatunensis* Poljakov ex U.P.Pratov & Bakanova, *A. albicaulis* Nevski, *A. algeriensis* Filatova, *A. amoena* Poljakov, *A. aralensis* Krasch., *A. araxina* Takht., *A. arenicola* Krasch. ex Poljakov, *A. assurgens* Filatova, *A. aucheri* Boiss., *A. badghysi* Krasch. & Lincz. ex Poljakov, *A. balchanorum* Krasch., *A. baldshuanica* Krasch. & Zopr., *A. barrelieri* Besser, *A. bashkalensis* Kursat & Civelek, *A. bicolor* Rech.f. & Wagenitz, *A. borotalensis* Poljakov, *A. brevifolia* Wall. ex DC., *A. caerulescens* L., *A. camelorum* Krasch., *A. chitralensis* Podlech, *A. cina* O.Berg, *A. ciniformis* Krasch. & Popov ex Poljakov, *A. compacta* Fisch. ex DC., *A. czukavinae* Filatova, *A. densiflora* Viv., *A. densifolia* Filatova, *A. diffusa* Krasch. ex Poljakov, *A. dubjanskyana* Krasch. ex Poljakov, *A. dumosa* Poljakov, *A. dzevanovskyi* Leonova, *A. elongata* Filatova & Ladygina, *A. eremophila* Krasch. & Butkov ex Poljakov, *A. fedorovii* Rzazade, *A. fedtschenkoana* Krasch., *A. ferganensis* Krasch. ex Poljakov, *A. finita* Kitag., *A. fragrans* Willd., *A. freitagii* Podlech, *A. fulvella* Filatova & Ladygina, *A. ghazniensis* Podlech, *A. ghoratensis* Podlech, *A. glanduligera* Krasch. ex Poljakov, *A. glaucina* Krasch. ex Poljak., *A. gracilescens* Krasch. & Iljin, *A. grenardii* Franch., *A. gurganica* (Krasch.) Filatova, *A. gypsacea* Krasch., Popov & Lincz. ex Poljakov, *A. halophila* Krasch., *A. heptapotamica* Poljakov, *A. herba-alba* Asso, *A. huguetii* Caball., *A. ifranensis* J.Didier, *A. inculta* Sieber ex DC., *A. issykkulensis* Poljakov, *A. kandaharensis* Podlech, *A. karatavica* Krasch. & Abolin ex Poljakov, *A. kasakorum* (Krasch.) Pavlov, *A. kaschgarica* Krasch., *A. kemrudica* Krasch., *A. kermanensis* Podlech, *A. knorringiana* Krasch., *A. kochiiformis* Krasch. & Lincz. ex Poljakov, *A. kopetdaghensis* Krasch., Popov & Lincz. ex Poljakov, *A. korovinii* Poljakov, *A. korshinskyi* Krasch. ex Poljakov, *A. kurramensis* Qazilb., *A. lehmanniana* Bunge, *A. lercheana* Weber ex Stechm., *A. lessingiana* Besser, *A. leucotricha* Krasch. ex Ladygina, *A. maritima* L., *A. minchunensis* (Y.R.Ling) G.H.Niu, B.H.Jiao & T.G.Gao, comb. nov., *A. mogoltavica* Poljakov, *A. mongolorum* Krasch., *A. mucronulata* Poljakov, *A. namanganica* Poljakov, *A. nigricans* Filatova & Ladygina, *A. nitrosa* Weber ex Stechm., *A. nutans* Willd., *A. oliveriana* J.Gay ex Besser, *A. oranensis* Deb. ex Filatova, *A.*

pauciflora Weber ex Stechmann, *A. pineticola* Kupr., *A. pineticola* Kupr., *A. porrecta* Krasch. ex Poljakov, *A. prolixa* Krasch. ex Poljakov, *A. qingheensis* G.Z.Jin, *A. quettensis* Podlech, *A. ramosa* C.Sm. ex Link, *A. rhodantha* Rupr., *A. saharae* Pomel, *A. saissanica* (Krasch.) Filatova, *A. santolina* Schrenk, *A. santonicum* L., *A. sawanensis* (Y.R.Ling & Humphries) G.H.Niu, B.H.Jiao & T.G.Gao, comb. nov. , *A. schrenkiana* Ledeb., *A. scopiformis* Ledeb., *A. scotina* Nevski, *A. semiarida* (Krasch. & Lavrenko) Filatova, *A. sieberi* Besser, *A. spicigera* K.Koch, *A. stenocephala* Krasch. ex Poljakov, *A. subchrysolepis* Filatova, *A. sublessingiana* Krasch. ex Poljakov, *A. subsalsa* Filatova, *A. szowitziana* (Besser) Grossh., *A. taurica* Willd., *A. tecti-mundi* Podlech, *A. tenuisecta* Nevski, *A. terrae-albae* Krasch., *A. thomsoniana* (C.B.Clarke) Filatova, *A. tianschanica* Krasch. ex Poljakov, *A. transiliensis* Poljakov, *A. turanica* Krasch., *A. turcomanica* Gand., *A. vachanica* Krasch. ex Poljakov, *A. valida* Krasch. ex Poljakov, *A. vallesiaca* All.

6 ***Artemisia*** subg. ***Tridentatae*** (Rydberg) McArthur, Amer. J. Bot. 68: 590. 1981. emend. B.H.Jiao & T.G.Gao, Ann. Bot. 131: 880. 2023. **Type**: *A. tridentata* Nutt.

Description: Subshrubs or shrubs, sometimes perennial herbs; 20–200 cm high. Root vertical. Stems numerous, sometimes few, erect. Leaves type 1 (trilobed small leaves, at least the basal leaves trilobed). Synflorescence panicle or raceme, rarely simple. Capitula *Seriphidium* or *Artemisia* type, also rarely *Absinthium* or *Dracunculus* type. Achenes without crown.

Species number and distribution: ca. 40 species; mainly distributed in western North America, with a few species in South America and Northeast Asia.

Notes: The original subg. *Tridentatae* sensu McArthur et al.[19] is characterized by discoid capitula and New World distribution. Based on our phylogenetic analysis[1] (Figure 2), we recognize an expanded *A.* subg. *Tridentatae* with the addition of *Sphaeromeria, Picrothamnus* from west North America, several South American species (e.g. *Artemisia sodiroi*), Beringian species (e.g. *A. furcata*), Northeast Asian species (e.g. *A. lagocephala*). All the species of newly included in *A.* subg. *Tridentatae* are shrubs or subshrubs, with basal leaves 3-lobed or entire.

6.1 ***Artemisia*** sect. ***Younghusbandianae*** B. H. Jiao & T. G. Gao, **sect. nov.** [member of subg. *Tridentatae*] **Type**: *A. younghusbandii* J. R. Drumm. ex Pamp.

Description: Shrubs or subshrubs, 15–30 cm high. Root vertical, with thick woody stock and branches, much branched; densely gray tomentose. Leaves type 3 (2-palmate medium leaves). Synflorescence panicle. Capitula *Absinthium* type. Disk florets corolla tubular; marginal florets corolla narrow tubular; style of disk florets bifid, apex truncate; style of marginal florets apex acute; anther apical appendage oblong; anther thecal base obtuse; anther collar oblong.

Species number and distribution: Only one species, *Artemisia younghusbandii*, endemic to Xizang, China.

Notes: *Artemisia younghusbandii* alone formed the most basal lineage of *A.* subg. *Tridentatae* (Figure 2). It has pubescent receptacles and was originally placed in *A.* subg. *Absinthium* sensu Ling et al. (2011) accordingly. Our phylogenetic analysis revealed it was firmly nested in *A.* subg. *Tridentatae* and closely related to *A. lagocephala* and other Beringian and New World species of *A.* subg. *Tridentatae*. Our examinations on its specimens revealed that its uppermost leaves are often 3-lobed, a character common in the *A.* subg. *Tridentatae* sensu McArthur et al.[19] and *A. lagocephala*. Its pubescent receptacle is also shared by *A. lagocephala.* However, we recognize that further morphological studies and increased sampling are necessary for a comprehensive understanding of this section.

Species included (1): *Artemisia younghusbandii* J.R.Drumm. ex Pamp.

6.2 ***Artemisia*** sect. ***Lagocephalae*** (Kitam.) B.H.Jiao & T.G.Gao, **stat. nov.** [member of subg.

Tridentatae] ≡ *Artemisia* ser. *Lagocephalae* Kitam. in Act. Phytotax. Geobot. 8: 64. 1939. **Type**: *A. lagocephala* (Fisch. ex Bess.) DC.

Description: Perennials or subshrubs, 40–60 cm high. Root vertical, with thick woody stock and branches, much branched; densely gray tomentose. Leaves type 1 (trilobed small leaves). Synflorescence raceme or panicle. Capitula *Absinthium* type. Disk florets corolla campanulate tubular; marginal florets corolla narrow tubular; style of disk florets bifid, apex truncate; style of marginal florets apex acute; anther apical appendage oblong; anther thecal base tailed; anther collar oblong or balusterform.

Species number and distribution: Three species. Distributed in Arctic, Siberia, and Far East regions of Asia.

Notes: We recognize *Artemisia* subg. *Tridentatae* sect. *Lagocephalae* by raising *A.* subg. *Artemisia* sect. *Absinthium* ser. *Lagocephalae* sensu Kitam to the rank of section and adding two species, i.e. *A. rutifolia* and *A. kruhsiana*. It is characterized by *Absinthium* type capitula (pubescent receptacles). The *Absinthium* type capitula is shared by most species of *A.* subg. *Absinthium*, but the species of this section share the same leave type (type 1) with other species of *A.* subg. *Tridentatae*.

Species included (3): *Artemisia kruhsiana* Besser, *A. lagocephala* (Fisch. ex Bess.) DC., *A. rutifolia* Stephan ex Spreng.

6.3 ***Artemisia*** sect. ***Tridentatae***. [member of subg. *Tridentatae*] **Type**: *A. tridentata* Nutt.

Description: Shrubs or subshrubs; 20–200 cm high. Root vertical, with thick woody stock and branches, much branched. Leaves type 1 (trilobed small leaves, or entire). Synflorescence raceme or panicle, rarely with only one capitulum. Capitula *Artemisia* or *Tridentatae* type, rarely *Dracunculus* type (*A. filifolia*). Disk florets corolla campanulate tubular, cup shaped tubular or tubular; marginal florets corolla narrow tubular; style of disk florets bifid, apex truncate or undivided; style of marginal florets apex acute or retuse; anther apical appendage oblong, acute or attenuate; anther thecal base obtuse, sagittate or tailed; anther collar oblong.

Species number and distribution: 36 species; western North America, the Arctic and temperate regions of South America.

Notes: *Artemisia* subg. *Tridentatae* sect. *Tridentatae* is the core of *A.* subg. *Tridentatae*. We expanded its circumscription by adding 27 species originally placed in *A.* subg. *Artemisia* or unplaced. The capitula type and synflorescence type seem diverse (Figure 4). We think the abundant new ecological opportunities provided when the ancestors of the sect. *Tridentatae* migrated to the New World may contribute to this. It seems that they all share the trilobed leaves. On the specimens of some species of this section (e.g. *Artemisia porteri* Cronquist), only entire leaves are present. However, its basal leaves are trilobed[20]. We acknowledge that further morphological work is required on these newly included species.

Species included (36): *Artemisia alaskana* Rydb., *A. albicans* Sòn.Garcia, Garnatje, McArthur, Pellicer, S.C.Sand, *A. arbuscula* Nutt., *A. bigelovii* A.Gray, *A. californica* Less., *A. cana* Pursh, *A. capitata* (Nutt.) Sòn.Garcia, Garnatje, McArthur, Pellicer, S.C.Sand. & Vallès-Xirau, *A. constricta* Sòn.Garcia, Garnatje, McArthur, Pellicer, S.C.Sand. & Vallès-Xirau, *A. copa* Phil., *A. echegarayi* Hieron., *A. filifolia* Torr., *A. franserioides* Greene, *A. furcata* M.Bieb., *A. globularia* Cham. ex Besser, *A. inaequifolia* Sòn.Garcia, Garnatje, McArthur, Pellicer, S.C.Sand. & Vallès-Xirau, *A. martirensis* (Wiggins) C.R.Hobbs & B.G.Baldwin, *A. mendozana* DC., *A. nesiotica* P.H.Raven, *A. nova* A.Nelson, *A. nuttallii* (Torr. & A.Gray) Mosyakin, L.M.Shultz & G.V.Boiko, *A. papposa* S.F.Blake & Cronquist, *A. pattersonii* A.Gray, *A. pedatifida* Nutt., *A. porteri* Cronquist, *A. potentilloides* A.Gray, *A. pygmaea*

A.Gray, *A. rigida* (Nutt.) A.Gray, *A. rothrockii* A.Gray, *A. ruthiae* (A.H.Holmgren, L.M.Shultz & Lowrey) Sòn.Garcia, Garnatje, McArthur, Pellicer, S.C.Sand. & Vallès_Xirau, *A. scopulorum* A.Gray, *A. simplex* (A.Nelson) Sòn.Garcia, Garnatje, McArthur, Pellicer, S.C.Sand. & Vallès-Xirau, *A. sodiroi* Hieron., *A. spiciformis* Osterh., *A. spinescens* D.C.Eaton, *A. tridentata* Nutt., *A. tripartita* Rydb.

7 ***Artemisia*** subg. ***Absinthium*** (Miller) Lessing, Syn. Gen. Compos. 264. 1832. emend. B. H. Jiao & T. G. Gao, Ann. Bot. 131: 880. 2023. **Type**: *A. absinthium* L.

Description: Annuals, biennials, perennials, or rarely shrubs; 20–150 cm high. Stems erect, prostrate, or rosette. Leaves show significant variations, including type 1, 2, 3, 4, 9, 10. Synflorescence panicle or raceme. Capitula *Absinthium* type, rarely *Seriphidium* type (sect. *Junceum*) or *Artemisia* type (sect. *Blepharolepides* and some other species). Achenes without crown and ribs.

Species number and distribution: ca. 63 species; mainly in the temperate or cold temperate regions of Eurasia, a few in North America.

Notes: Previous *A.* subg. *Absinthium* was characterized by *Absinthium* type capitula with pubescent receptacles, but whether pubescent receptacle is a good taxonomic character has always been questioned[21]. Jiao et al.[1] reconstructed the evolution pattern of capitula type, and revealed that *Absinthium* type capitula has multiple origins in *Artemisia*. Based on these, we modified the circumscription of *A.* subg. *Absinthium* by excluding short annual halophytes (*A.* subg. *Seriphidium* sect. *Anethifoliae*), and shrubby species with 3-lobed or pinnatisect leaves (*A.* subg. *Tridentatae* sect. *Younghusbandianae* and sect. *Lagocephalae*), and adding annual species with *Seriphidium* type capitula (sect. *Juncea*), and an annual species with *Artemisia* type capitula (sect. *Blepharolepides*). The *Absinthium* type capitula, in conjunction with globose capitula and 2-palmate medium leaves, can be utilized to define the recircumscribed *A.* subg. *Absinthium*. Despite its limited species number, this subgenus boasts the broadest ecological adaptation range. It encompasses, for instance, the common temperate weed *A. sieversiana*, the Eurasian grassland dominant species *A. frigida*, as well as the drought-resistant *A. xerophytica* that thrives in arid deserts, the cushionplant *A. minor* that adapts to alpine habitats, and the island species *A. thuscula*. Consequently, *A.* subg. *Absinthium* constitutes an excellent taxon for investigating adaptive evolution.

7.1 ***Artemisia*** sect. ***Blepharolepides*** (Y. R. Ling) B.H.Jiao & T.G.Gao, **stat. nov.** [member of subg. *Absinthium*] ≡ *Artemisia* ser. *Blepharolepides* Y. R. Ling in Bull. Bot. Res. 8(4): 54. 1988. **Type**: *A. blepharolepis* Bge.

Description: Annuals or biennials; 20–60 cm high. Root vertical. Stems few, many branches, erect. Leaves type 4 (small, 2-pinnatisect, multiple-lobed). Synflorescence panicle. Capitula *Artemisia* type; corolla dark yellow. Disk florets corolla cup shaped tubular; marginal florets corolla narrow tubular; style of disk florets bifid, apex truncate; style of marginal florets apex retuse; anther apical appendage acute; anther thecal base sagittate; anther collar balusterform.

Species number and distribution: Only one species; Northern China and Mongolia.

Notes: *Artemisia blepharolepis* was previously placed within the *A.* subg. *Dracunculus*[13] as its disk florets were regarded as functionally staminate. We checked its specimens and wild plants, and confirmed that its disk florets are bisexual and fertile. Furthermore, our nuclear phylogeny strongly supported it is not a member of *A.* subg. *Dracunculus*, instead of the basal lineage of *A.* subg. *Absinthium* (Figure 2). Unlike the original *A.* subg. *Absinthium* sensu Lessing, its receptacles are glabrous, not pubescent. Our analysis on the evolution of capitula type indicated that pubescent receptacles have evolved multiple times across the genus *Artemisia*. This species is easy to identify due to its relatively narrow synflorescence, narrow and multi lobed leaves, and the fast-growing annual

habit. It usually germinates and grows rapidly after summer rain, and then flowers and fruits until death in short time.

Species included (1): *Artemisia blepharolepis* Bunge.

7.2 ***Artemisia*** sect. ***Sieversianae*** Wendelb. in Biblioth. Bot. 125: 49. 1960. [member of subg. *Absinthium*] **Type**: *A. sieversiana* Ehrhart ex Willd.

Description: Annuals or biennials. Root vertical. Stems single, erect, more than 100 cm high; or few, prostrate, 15–30 cm high. Leaves type 3 (small 2-palmate medium leaves), and type 10 (large, pinnatisect broad-lobed). Synflorescence panicle. Capitula *Absinthium* or rarely *Artemisia* type (*A. shangnanensis*), oblate or globose, 5–10 mm in diam. Disk florets corolla campanulate tubular; marginal florets corolla conical; style of disk florets bifid, apex truncate; style of marginal florets apex retuse; anther apical appendage acute, attenuate or oblong; anther thecal base obtuse or sagittate; anther collar balusterform.

Species number and distribution: ca. seven species; *Artemisia sieversiana* widely distributed in temperate Asia, others in Central Asia and Northern Asia.

Notes: *Artemisia* subg. *Absinthium* sect. *Sieversianae* is characterized by having big globose capitula. *A. succulentoides* fits well morphologically in this section. However, only the type collections of *A. succulentoides* are available. Despite two explicit searches for it in its type locality (Xizang, China), we have been unsuccessful. Consequently, the taxonomic position of this species necessitates further investigation.

Species included (7): *Artemisia jacutica* Drobow, *A. macrocephala* Jacquem. ex Besser, *A. pallens* Wall. ex DC., *A. samoiedorum* Pamp., *A. shangnanensis* Ling & Y.R.Ling, *A. sieversiana* Ehrh. ex Willd., *A. succulenta* Ledeb.

7.3 ***Artemisia*** sect. ***Absinthium***. [member of subg. *Absinthium*] **Type**: *A. absinthium* L.

Description: Perennials, shrubs or subshrubs. Root vertical. Stems single or few, erect, more than 100 cm high. Leaves type 9 (medium to big, pinnatisect, lobe ovate). Synflorescence panicle. Synflorescence panicle. Capitula *Absinthium* type, oblate or globose. Disk florets corolla campanulate tubular; marginal florets corolla conical; style of disk florets bifid, apex truncate; style of marginal florets apex acute; anther apical appendage acute; anther thecal base Sagittate or obtuse; anther collar balusterform.

Species number and distribution: five species; *Artemisia absinthium* widely distributed in Central Asia, Europe, and North Africa, *A. arborescens* in the Mediterranean area, the other three species endemic to Atlantic islands, including *A. thuscula* from the Canary Islands, *A. argentea* from the Madeira Islands and *A. gorgonum* from Cape Verde.

Notes: *Artemisia* subg. *Absinthium* sect. *Absinthium* include only *A. absinthium* and its close relatives. Sect. *Absinthium* and sect. *Sieversianae* are sister groups. They all have large, globose capitula. The difference is that sect. *Absinthium* is a shrubby group, mainly distributed in the west end of the Eurasian continent, while all sect. *Sieversianae* species are annual herbs, mainly distributed in the east end of the Eurasian continent. Interestingly, there are 3 Atlantic islands endemic species in sect. *Absinthium.* Our phylogenetic analysis showed that *A. absinthium* was resolved at the base of this clade, followed by the three Atlantic island endemic species (Figure 2). *A. arborescens* (distributed around the Mediterranean) and *A. thuscula* (endemic to Canary Islands) is a sister group, which forms the latest diverging lineage of this clade.

Species included (5): *Artemisia absinthium* L., *A. arborescens* L., *A. argentea* L'Hér., *A. gorgonum* Webb, *A. thuscula* Cav.

7.4 ***Artemisia*** sect. ***Juncea*** Poljakov ex Filatova, Novosti Sist. Vyssh. Rast. 23: 219. 1986. [member of subg. *Absinthium*] **Type**: *A. juncea* Kar. & Kir.

Description: Annuals, or perennials. Stem numerous, erect, 30–60 cm high. Leaves type 1 (trilobed small leaves, or entire). Synflorescence panicle. Capitula *Seriphidium* type, oblong. Disk florets corolla cup shaped tubular; marginal florets corolla absent; style of disk florets bifid, apex truncate; style of marginal florets absent; anther apical appendage oblong; anther thecal base obtuse; anther collar oblong.

Species number and distribution: four species; Central Asia.

Notes: *Artemisia* subg. *Absinthium* sect. *Juncea* is characterized by having *Seriphidium* type capitula and entire or 3-lodeb leaves. It was once placed in *A.* subg. *Seriphidium* sensu Poljakov[12], but the species of this section and the original *A.* subg. *Seriphidium* (sect. *Seriphidium* in present study) can be distinguished by leaf type (type 1 in this section vs type 2 in sect. *Seriphidium*). Our phylogenetic analysis firmly placed this section in the *A.* subg. *Absinthium* (Figure 2).

Species included (4): *Artemisia deserti* Krasch., *A. juncea* Kar. & Kir., *A. leucodes* Schrenk, *A. macrosciadia* Poljakov.

7.5 ***Artemisia*** sect. ***Argyrophyllae*** Poljak., Fl. USSR 26: 522. 1961. [member of subg. *Absinthium*] **Type**: *Artemisia argyrophylla* Ledeb.

Description: Perennials, rarely annuals or shrubs. Stem numerous, erect, 10–40 cm high, tomentose or puberulent. Leaves type 3 (2-palmate small to medium leaves), rarely type 2 (small, pinnatisect multiple-lobed). Synflorescence panicle or raceme, rarely single. Capitula *Absinthium* or rarely *Artemisia* type; ovoid or oblate. Disk florets corolla campanulate tubular; marginal florets corolla narrow tubular; style of disk florets bifid, apex truncate; style of marginal florets apex acute; anther apical appendage oblong or acute; anther thecal base sagittate, tailed or obtuse; anther collar oblong or balusterform.

Species number and distribution: ca. 47 species; North America, South America and Eurasia.

Notes: *Artemisia* subg. *Absinthium* sect. *Argyrophyllae* is the core of *A.* subg. *Absinthium.* It is characterized by ovoid capitula, 2-palmate small to medium leaves, and plant densely covered with hairs. Many species within this section may be among the most adapted to harsh alpine habitats within the genus *Artemisia*, as they are short in plant height, densely covered with hairs, and having short, narrow synflorescences. Mas de Xaxars et al.[22] reported that the chromosome base number of the Alpine species of this section changed from ancestral state x = 9 to x = 8, and the most reported chromosome numbers in sect. *Argyrophyllae* are diploid, with only a few species being polyploid. It is possible that changes in the chromosome base number, rather than polyploidization, play a significant role in the adaptation of sect. *Argyrophyllae* to the harsh alpine habitats.

Species included (47): *Artemisia abbreviata* (Krasch. ex Korobkov) Krasnob., *A. aleutica* Hultén, *A. alpina* Pall. ex Willd., *A. argyrophylla* Ledeb., *A. aschurbajewi* C.Winkl., *A. assoana* Willk., *A. atlantica* Coss. & Durieu, *A. austriaca* Jacq., *A. caespitosa* Ledeb., *A. cuspidata* Krasch., *A. czekanowskiana* Trautv., *A. davazamczii* Darijma & Kamelin, *A. disjuncta* Krasch., *A. filatovae* Kupr., *A. frigida* Willd., *A. genipi* Stechm., *A. glacialis* L., *A. glomerata* Ledeb., *A. granatensis* Boiss., *A. haussknechtii* Boiss., *A. hippolyti* A.Butkov, *A. incana* Druce, *A. judaica* L., *A. kitadakensis* Hara & Kitam., *A. lagopus* Fisch. ex Besser, *A. aethiopica* L., *A. melanolepis* Boiss., *A. minor* Jacquem. ex Besser, *A. nepalensis* Nees, *A. nitida* Bertol., *A. nivalis* Braun-Blanq., *A. obtusiloba* Ledeb., *A. pedemontana* Balb., *A. penicilliformis* (Shih) M. Wei & T. G. Gao, *A. radicans* Kupr., *A. reptans* C.Sm., *A. rupestris* L., *A. schmidtiana* Maxim., *A. senjavinensis* Bess, *A. sericea* Weber ex Stechm., *A.*

skorniakovii C.Winkl., *A. splendens* Willd., *A. succulentoides* Ling & Y.R.Ling, *A. umbelliformis* Lam., *A. viridis* Willd. ex DC., *A. woodii* (Neilson) C.W.Riggins, *A. xerophytica* Krasch.

8 ***Artemisia*** subg. ***Artemisia*. Type**: *A. vulgaris* L.

Description: Perennials. Stems erect; 20–150 cm high. Leaves large, type 10 (medium to large, pinnatisect broad-lobed), rarely Type 7 (Entire medium leaves, but not toothed), type 8 (large, 5-lobed to pinnatipartite); if not entire, the width of lobes > 2 mm. Synflorescence panicle or raceme. Capitula *Artemisia* type. Achenes without crown and ribs.

Species number and distribution: ca. 111 species; mainly in East Asia, a few extending to Europe and New World.

Notes: Subgenus *Artemisia* is the only group in the genus that prefers wet habitats. The previous *A.* subg. *Artemisia* is characterized by receptacles glabrous, capitula disciform, disk florets bisexual and fertile. This type of capitula, called "*Artemisia* type" here, represents the ancestral state of the whole genus (Supplementary Figure 12). Therefore, all species with *Artemisia* type capitula were assigned into this group without phylogenetic evidence by many authors[12,13,23]. Here, based on the morphological and phylogenetic evidences, we recognize this diminished *A.* subg. *Artemisia* by transferring species to *A.* subg. *Dracunculus*, *A.* subg. *Pectinatae*, *A.* subg. *Ponticae*, *A.* subg. *Seriphidium*, and *A.* subg. *Tridentatae.* There are significant differences in the phylogenetic results between the nuclear genes and chloroplast genomes. The phylogeny of chloroplast data showed the *A.* subg. *Artemisia* is not monophyletic, but divided into two clades (Figure 3), corresponding to *A.* subg. sect. *Selengenses* and *A.* subg. sect. *Artemisia* respectively (Figure 2).

8.1 ***Artemisia*** sect. ***Selengenses*** (Pamp.) B.H.Jiao & T.G.Gao, **stat. nov.** [member of subg. *Artemisia*]≡*Artemisia* subsect. *Selengenses* Pamp. in Nuov. Giorn. Bot. Ital. n.s. 36: 500. 1930. **Type**: *A. selengensis* Turcz. ex Besser

Description: Perennial herbs, sometimes subshrubs; 70–120 cm high; rootstock horizontally creeping to obliquely rising. Leaves medium to large, type 7 (entire leaves, but not toothed), or Type 8 (5-lobed to pinnatipartite). Synflorescence panicle, rarely raceme. Capitula *Artemisia* type, oblong, ovoid, or ovoid-campanulate; 2–5 mm in diam. Disk florets corolla cup shaped tubular; marginal florets corolla filiform; style of disk florets bifid, apex truncate; style of marginal florets apex acute; anther apical appendage attenuate; anther thecal base tailed or sagittate; anther collar balusterform.

Species number and distribution: ca. 14 species; Northeast Asia and North America.

Notes: The recircumscribed *Artemisia* subg. *Artemisia* sect. *Selengenses* was raised from the original *Artemisia* subg. *Artemisia* sect. *Artemisia* ser. *Selengenses* sensu Ling et al.[13]. It was much expanded by including some East Asian species such as *A. anomala* and *A. viridissima*, and some North American species such as *A. ludoviciana* (= *A. vulgaris* sensu Keck) complex[21,24].

Species included (14): *Artemisia amygdalina* Decne., *A. anomala* S.Moore, *A. carruthii* Alph.Wood ex J.H.Carruth, *A. deversa* Diels, *A. douglasiana* Besser, *A. longifolia* Nutt., *A. ludoviciana* Nutt., *A. michauxiana* Besser, *A. packardiae* J.W.Grimes & Ertter, *A. selengensis* Turcz. ex Besser, *A. serrata* Nutt., *A. suksdorfii* Piper, *A. tilesii* Ledeb., *A. viridissima* Pamp.

8.2 ***Artemisia*** sect. ***Artemisia*** [member of subg. *Artemisia*] **Type**: *A. vulgaris* L.

Description: Perennial herbs, sometimes subshrubs; 30–150 cm high; rootstock horizontally creeping to obliquely rising. Leaves large, type 10 (pinnatisect broad-lobed), rarely type 7 (entire leaves, but not toothed), type 8 (5-lobed to pinnatipartite). Synflorescence panicle, rarely raceme; capitula *Artemisia* type; oblong, ovoid, or ovoid-campanulate; 2–5 mm in diam. Disk florets corolla cup shaped tubular; marginal florets corolla filiform; style of disk florets bifid, apex truncate; style of marginal florets apex

acute; anther apical appendage attenuate or acute; anther thecal base tailed or sagittate; anther collar balusterform, rare oblong.

Species number and distribution: ca. 97 species; widely distributed in the temperate regions of Eurasia, a few extending into the tropical regions of Southeast Asia.

Notes: The recircumscribed *Artemisia* subg. *Artemisia* sect. *Artemisia* is the core of *A.* subg. *Artemisia*. It is roughly equivalent to the sum of the sect *Artemisia*, sect. *Albibractea*, and sect. *Viscidipubes* sensu Ling et al.[13]. These sections were characterized by their white phyllary (sect. *Albibractea*) and glandular pubescence (sect. *Viscidipubes*). However, the monophyly of these sections was not supported in our study (Figure 2). Therefore, it can be inferred that phyllary color, hair type can't be used as taxonomic characters for section level in *A.* subg. *Artemisia*. Natural hybridization among species within this section, such as *A. gilvescens* and *A. indica* var. *maximowiczii*[25], has been documented. Concurrently, all species of *A.* subg. *Artemisia* sect. *Artemisia* possess well-developed rhizomes and the ability to reproduce asexually. This enables rapid fixation and dissemination of mutations. Although our phylogenetic analysis indicates that this group can be bifurcated into two subgroups (Figure 2), no distinct morphological characteristics exist to further differentiate them. Consequently, additional morphological research and increased sampling are necessary to categorize them into natural groups.

Species included (100): *Artemisia abaensis* Y.R.Ling & S.Y.Zhao, *A. argyi* H.Lév. & Vaniot, *A. atrovirens* Hand.-Mazz., *A. austrohimalayaensis* Y.R.Ling & Puri, *A. austroyunnanensis* Ling & Y.R.Ling, *A. banihalensis* M.K.Kaul & S.K.Bakshi, *A. bhutanica* Grierson & Spring., *A. brachyphylla* Kitam., *A. calophylla* Pamp., *A. campbellii* Hook.f. & Thomson ex C.B.Clarke, *A. chingii* Pamp., *A. codringtonii* Rech.f., *A. comaiensis* Ling & Y.R.Ling, *A. divaricata* (Pamp.) Pamp., *A. domingensis* Urb., *A. emeiensis* Y.R.Ling, *A. eriocephala* Pamp., *A. erlangshanensis* Y. Ling & Y. R. Ling, *A. estesii* K.L.Chambers, *A. flaccida* Hand.-Mazz., *A. fulgens* Pamp., *A. gilvescens* Miq., *A. gongshanensis* Y.R.Ling & Humphries, *A. gyitangensis* Ling & Y.R.Ling, *A. hanwulaensis* Y.Z.Zhao, *A. igniaria* Maxim., *A. imponens* Pamp., *A. incisa* Pamp., *A. indica* Willd., *A. integrifolia* L., *A. javanica* Pamp., *A. jilongensis* Y.R.Ling & Humphries, *A. kanashiroi* Kitam., *A. kawakamii* Hayata, *A. koidzumii* Nakai, *A. lactiflora* Wall. ex DC., *A. lanaticapitula* X. F. Jin, Z. H. Chen & Y. F. Lu, *A. lancea* Vaniot, *A. leucophylla* C.B.Clarke, *A. lingyeouruennii* L.M.Shultz & Boufford, *A. mattfeldii* Pamp., *A. mongolica* (Fisch. ex Besser) Nakai, *A. monophylla* Kitam., *A. montana* (Nakai) Pamp., *A. moorcroftiana* Wall. ex DC., *A. myriantha* Wall. ex Besser, *A. nepalica* Yonek., *A. nilagirica* (C.B.Clarke) Pamp., *A. nujianensis* (Ling & Y.R.Ling) Y.R.Ling, *A. occidentalisichuanensis* Y.R.Ling & S.Y.Zhao, *A. occidentalisinensis* Y.R.Ling, *A. orientalihengduangensis* Ling & Y.R.Ling, *A. orientalixizangensis* Y.R.Ling & Humphries, *A. orientaliyunnanensis* Y.R.Ling, *A. palmeri* A.Gray, *A. phyllobotrys* (Hand.-Mazz.) Ling & Y.R.Ling, *A. princeps* Pamp., *A. pringlei* Greenm., *A. qinlingensis* Ling & Y.R.Ling, *A. robusta* (Pamp.) Ling & Y.R.Ling, *A. rosthornii* Pamp., *A. roxburghiana* Besser, *A. rubripes* Nakai, *A. saitoana* Kitam., *A. shennongjiaensis* Ling & Y.R.Ling, *A. sichuanensis* Ling & Y.R.Ling, *A. simulans* Pamp., *A. neosinensis* B. H. Jiao & T. G. Gao, *A. smithii* Mattf., *A. somae* Hayata, *A. speciosa* (Pamp.) Ling & Y.R.Ling, *A. stelleriana* Besser, *A. stipularis* Urb. & Ekman, *A. stolonifera* (Maxim.) Kom., *A. subulata* Nakai, *A. sylvatica* Maxim., *A. tafelii* Mattf., *A. tainingensis* Hand.-Mazz., *A. tangutica* Pamp., *A. thellungiana* Pamp., *A. tsuneoi* Tatew. & Kitam., *A. tukuchaensis* Kitam., *A. umbrosa* (Besser) Turcz. ex Verl., *A. velutina* Pamp., *A. verbenacea* (Kom.) Kitag., *A. verlotiorum* Lamotte, *A. vexans* Pamp., *A. viridisquama* Kitam., *A. viscida* Pamp., *A. viscidissima* Ling & Y.R.Ling, *A. vulgaris* L., *A. yadongensis* Ling & Y.R.Ling, *A. yongii* Y.R.Ling, *A. yunnanensis*

Jeffrey, *A. zayuensis* Ling & Y.R.Ling, *A. zhongdianensis* Y.R.Ling.

Supplementary References

1. Jiao, B. *et al.* Phylogenomics and morphological evolution of the mega-diverse genus *Artemisia* (Asteraceae: Anthemideae): implications for its circumscription and infrageneric taxonomy. *Ann. Bot.* **131**, 867-883 (2023).
2. Yu, Y., Harris, A.J., Blair, C. & He, X. RASP (Reconstruct Ancestral State in Phylogenies): A tool for historical biogeography. *Mol. Phylogenet. Evol.* **87**, 46-49 (2015).
3. Shimodaira, H. & Hasegawa, M. Multiple comparisons of log-likelihoods with applications to phylogenetic inference. *Mol. Biol. Evol.* **16**, 1114-1116 (1999).
4. Kishino, H. & Hasegawa, M. Evaluation of the maximum likelihood estimate of the evolutionary tree topologies from DNA sequence data, and the branching order in Hominoidea. *J. Mol. Evol.* **29**, 170-179 (1989).
5. Shimodaira, H. An approximately unbiased test of phylogenetic tree selection. *Syst. Biol.* **51**, 492-508 (2002).
6. Rice, A. *et al.* The Chromosome Counts Database (CCDB) - a community resource of plant chromosome numbers. *New Phytol.* **206**, 19-26 (2015).
7. White, T.J., Bruns, T., Lee, S., & Taylor, J. Amplification and direct sequencing of fungal ribosomal RNA genes for phylogenetics. in *PCR Protocols: A Guide to Methods and Applications* (ed. Innis, M., Gelfand, D., Sninsky, J., & White, T.) 315-322 (Academic Press, San Diego, 1990).
8. Markos, S., & Baldwin, B. G. Higher-level relationships and major lineages of Lessingia (Compositae, Astereae) based on nuclear rDNA internal and external transcribed spacer (ITS and ETS) sequences. *Syst. Bot.* **26**, 168-183 (2001).
9. Baldwin, B.G., & Markos, S. Phylogenetic utility of the external transcribed spacer (ETS) of 18S–26S rDNA: Congruence of ETS and ITS trees of Calycadenia (Compositae). *Mol. Phylogenet. Evol.* **10**, 449-463 (1998).
10. Shultz, L.M. Monograph of *Artemisia* Subgenus *Tridentatae* (Asteraceae-Anthemideae. *Syst. Bot. Monogr.* **89**, 1-131 (2009).
11. Pellicer, J., Valles, J., Korobkov, A.A. & Garnatje, T. Phylogenetic relationships of *Artemisia* subg. *Dracunculus* (Asteraceae) based on ribosomal and chloroplast DNA sequences. *Taxon* **60**, 691-704 (2011).
12. Poljakov, P.P. *Artemisia* L. in *Flora of the U.S.S.R.*, Vol. 26 (ed. Shishkin, B.K., & Bobrov, E.G.) 425-631 (Nauka, Leningrad 1961).
13. Ling, Y.R., Humphries, C.J. & Gilbert, M.G. *Artemisia* L. in *Flora of China*, Vol. 20 (ed. Wu, Z.Y., Raven, P.H. & Hong, D.Y.) 1151-1259 (Science Press, Missouri Botanical Garden Press, Beijing, St. Louis, 2011).
14. Pellicer, J. *et al.* Genome size dynamics in *Artemisia* L. (Asteraceae): following the track of polyploidy. *Plant Biol.* **12**, 820-830 (2010).
15. Garcia, S. *et al.* Variation of DNA amount in 47 populations of the subtribe Artemisiinae and related taxa (Asteraceae, Anthemideae): karyological, ecological, and systematic implications. *Genome* **47**, 1004-1014 (2004).
16. Hobbs, C.R. & Baldwin, B.G. Asian origin and upslope migration of Hawaiian *Artemisia* (Compositae-Anthemideae). *J. Biogeogr.* **40**, 442-454 (2013).
17. Tkach, N.V., Hoffmann, M.H., Roser, M., Korobkov, A.A. & von Hagen, K.B. Parallel evolutionary patterns in multiple lineages of arctic *Artemisia* L. (Asteraceae). *Evolution* **62**, 184-198 (2008).

18. Safronova, I.N. Species of *Artemisia* subgenus *Seriphidium* in the West Turan and their ecology. in *Compositae: Biology & Utilization. Proceedings of the International Compositae Conference, Kew, 1994*, Vol. 2 (ed. Caligari, P.D.S., & Hind, D.J.N.) 105-110 (Royal Botanic Gardens, Kew, 1996).
19. McArthur, E., Pope, C. & Freeman, D. Chromosomal studies of subgenus *Tridentatae* of *Artemisia*: evidence for autopolyploidy. *Am. J. Bot.* **68**, 589-605 (1981).
20. Cronquist, A. A New *Artemisia* from Wyoming. *Madroño* **11**, 145-146 (1951).
21. Riggins, C.W. & Seigler, D.S. The genus *Artemisia* (Asteraceae: Anthemideae) at a continental crossroads: molecular insights into migrations, disjunctions, and reticulations among Old and New World species from a Beringian perspective. *Mol. Phylogenet. Evol.* **64**, 471-490 (2012).
22. Mas de Xaxars, G. *et al.* Impact of dysploidy and polyploidy on the diversification of high mountain *Artemisia* (Asteraceae) and allies. *Alp. Bot.* **126**, 35-48 (2016).
23. Tutin, T.G. *Artemisia* L. in *Flora Europaea*, Vol. 4 (ed. Tutin, T.G. *et al.*) 178-186 (Cambridge University Press, Cambridge, 1976).
24. Keck, D.D. A revision of the *Artemisia* vulgaris complex in North America. *Proc. Calif. Acad. Sci.* **25**, 421-468 (1946).
25. Yamashiro, T., Ogawa, M., Yamashiro, A. & Maki, M. Natural Hybridization between the Endangered Herb *Artemisia gilvescens* (Asteraceae) and the Common Congener, *Artemisia indica* var. *maximowiczii* in Japan. *Acta Phytotax. Geo Bot.* **69**, 109-117 (2018).
