# Supplementary material for: Global phylogeny and taxonomy of Artemisia
Source: Nat Commun. 2025 Oct 8;16:8648. doi: 10.1038/s41467-025-64039-0 (PMC12508166; doi:10.1038/s41467-025-64039-0)
Supplement: Supplementary file 3 — Description of Additional Supplementary Files [file 41467_2025_64039_MOESM3_ESM.pdf]

Description of Additional Supplementary Files

Supplementary Data 1. Phylogenetic studies and historical taxonomic resources for *Artemisia*.

Supplementary Data 2. Taxa included in this study, with details on sampling latitude/longitude, herbarium information, material type, and GenBank accession numbers.

Supplementary Data 3. Qualitative macromorphological character states of *Artemisia* and its allies.

Supplementary Data 4. Quantitative macromorphological character states of *Artemisia* and its allies.

Supplementary Data 5. Micromorphological character states of *Artemisia* and its allies.

Supplementary Data 6. Comprehensive species list of *Artemisia*, showing subgeneric and sectional positions with related evidence, and comparison of accepted names between this study and Plants of the World Online (POWO, https://powo.science.kew.org/), Global Compositae Checklist (GCC, https://www.compositae.org/), The World Flora Online (WFO, https://www.worldfloraonline.org/), and the Catalogue of Life (COL, https://www.catalogueoflife.org/), retrieved 29 September 2024.
