# Supplementary material for: Global phylogeny and taxonomy of Artemisia
Source: Nat Commun. 2025 Oct 8;16:8648. doi: 10.1038/s41467-025-64039-0 (PMC12508166; doi:10.1038/s41467-025-64039-0)
Supplement: Supplementary file 10 — Reporting Summary [file 41467_2025_64039_MOESM10_ESM.pdf]

nature portfolio

Corresponding author(s): Tiangang Gao

Last updated by author(s): 28/7/25

Reporting Summary

Nature Portfolio wishes to improve the reproducibility of the work that we publish. This form provides structure for consistency and transparency in reporting. For further information on Nature Portfolio policies, see our Editorial Policies and the Editorial Policy Checklist.

Statistics

For all statistical analyses, confirm that the following items are present in the figure legend, table legend, main text, or Methods section.

n/a	Confirmed	
☐	☒	The exact sample size (*n*) for each experimental group/condition, given as a discrete number and unit of measurement
☐	☒	A statement on whether measurements were taken from distinct samples or whether the same sample was measured repeatedly
☐	☒	The statistical test(s) used AND whether they are one- or two-sided<br>*Only common tests should be described solely by name; describe more complex techniques in the Methods section.*
☐	☒	A description of all covariates tested
☐	☒	A description of any assumptions or corrections, such as tests of normality and adjustment for multiple comparisons
☐	☒	A full description of the statistical parameters including central tendency (e.g. means) or other basic estimates (e.g. regression coefficient) AND variation (e.g. standard deviation) or associated estimates of uncertainty (e.g. confidence intervals)
☐	☒	For null hypothesis testing, the test statistic (e.g. *F*, *t*, *r*) with confidence intervals, effect sizes, degrees of freedom and *P* value noted<br>*Give P values as exact values whenever suitable.*
☒	☐	For Bayesian analysis, information on the choice of priors and Markov chain Monte Carlo settings
☐	☒	For hierarchical and complex designs, identification of the appropriate level for tests and full reporting of outcomes
☒	☐	Estimates of effect sizes (e.g. Cohen's *d*, Pearson's *r*), indicating how they were calculated

Our web collection on statistics for biologists contains articles on many of the points above.

Software and code

Policy information about availability of computer code

Data collection	DNA data were obtained from two sources: direct sequencing and extraction from GenBank. Macromorphological data were collected through direct observations in natural habitats, examination of specimens, and review of the published literature. Micromorphological data were gathered from samples collected in natural environments and herbarium specimens.
Data analysis	The scripts used for discretization continuous morphological character are available at https://doi.org/10.6084/m9.figshare.28164335. We used the following software packages: Trinity v2013-11-10, GoldFinder (https://bitbucket.org/oscarvargash/goldfinder/), MarkerMiner v1.2, SeqyClean v1.10.09, BBTools v38.90, sam2consense v2.0, python script prank_wrapper. py and phyutility_wrapper.py (https://bitbucket.org/yangya/phylogenomic_dataset_construction), GetOrganelle v1.7.6.1, GeSeq v2.03, tRNAscan-SE v2.0.733, Geneious v11.0.4, MAFFT v7.2234, BioEdit v.7.0.5.3, IQ-TREE v.2.0.6, ASTRAL III v.5.7.4, Newick Utilities v1.6, PhyloNet v.3.6.9, SplitsTree4 V4.16.1, RASP v4.2, and the R package "ape" v5.6-2.

For manuscripts utilizing custom algorithms or software that are central to the research but not yet described in published literature, software must be made available to editors and reviewers. We strongly encourage code deposition in a community repository (e.g. GitHub). See the Nature Portfolio guidelines for submitting code & software for further information.

Data

Policy information about availability of data

All manuscripts must include a data availability statement. This statement should provide the following information, where applicable:

- Accession codes, unique identifiers, or web links for publicly available datasets
- A description of any restrictions on data availability
- For clinical datasets or third party data, please ensure that the statement adheres to our policy

All data generated or analysed in this study are included in this published article and/or its supplementary materials. Sequencing data generated in this study have been deposited in GenBank under Bioproject PRJNA909040. All sequences used in the phylogenetic analyses are available in GenBank, with accession numbers listed in Supplementary Data 2. Morphological character data generated in this study is provided in Supplementary Data 3-5. The sectional taxonomy of Artemisia with an identification key to all the sections is provided in Supplementary Note 1. The accepted species list of Artemisia with subgeneric, sectional positions (including supporting evidence), and comparisons with databases and previous taxonomies are provided in Supplementary Data 6. Phylogenies and datasets generated during and/or analysed in this study have been deposited in figshare using https://doi.org/10.6084/m9.figshare.28164335).

Research involving human participants, their data, or biological material

Policy information about studies with human participants or human data. See also policy information about sex, gender (identity/presentation), and sexual orientation and race, ethnicity and racism.

Reporting on sex and gender	Not applicable to this study
Reporting on race, ethnicity, or other socially relevant groupings	Not applicable to this study
Population characteristics	Not applicable to this study
Recruitment	Not applicable to this study
Ethics oversight	Not applicable to this study

Note that full information on the approval of the study protocol must also be provided in the manuscript.

Field-specific reporting

Please select the one below that is the best fit for your research. If you are not sure, read the appropriate sections before making your selection.

☐ Life sciences ☐ Behavioural & social sciences ☒ Ecological, evolutionary & environmental sciences

For a reference copy of the document with all sections, see nature.com/documents/nr-reporting-summary-flat.pdf

Ecological, evolutionary & environmental sciences study design

All studies must disclose on these points even when the disclosure is negative.

Study description	We reconstructed the most comprehensive phylogeny of Artemisia to date, using genomic data and a gigamatrix approach. We also analyzed evolutionary patterns of 20 morphological characters of Artemisia worldwide to evaluate their taxonomic utility. Based on these findings, we propose a new global taxonomy for Artemisia, recognizing 8 subgenera and 24 sections, with taxonomic placements for 502 of the 505 accepted species.
Research sample	Nine diploid species, representing all eight Artemisia subgenera and one outgroup, were sequenced to obtain transcriptome data for selecting low-copy nuclear genes. Genome-skimming data were obtained for 314 species (298 Artemisia + 16 allied species) to capture nuclear low-copy genes, plastomes, and ITS/ETS regions. The final gigamatrix includes 414 species (394 Artemisia species and 20 outgroups from 13 related genera). Macromorphological data were collected for 394 Artemisia species through direct observations in natural habitats, examination of specimens, and review of the published literature. Micromorphological data were obtained for 200 Artemisia species, utilizing samples collected in natural environments and herbarium specimens.
Sampling strategy	We sampled 394 species (one individual per species), encompassing all eight recognized subgenera (78% of accepted diversity). Outgroups comprised 20 species from 13 closely related genera within tribe Anthemideae, selected via the latest phylogenetic framework. While no formal sample size calculation was performed, this taxon coverage ensures robust representation of intraspecific and intergeneric variation critical for our comparative analyses.
Data collection	Samples for DNA analysis were collected during on-site expeditions by BHJ, MW, CC, JYZ, JHS, FV, and TGG; herbarium samples were collected by BHJ, MW, CC, JYZ, DV, FV, ASE, APS, AS, XJG, FJ, AL, GK, and TGG. Morphological study of the herbarium specimens was conducted by BHJ, TGG, GHN, WW, and GYH.
Timing and spatial scale	DNA data was downloaded from GenBank on July 17, 2023. The Plants of the World Online and the Global Compositae Checklist were accessed on September 29, 2024. Morphological data were collected continuously from 2015 to 2025, with ongoing sampling/

	observations. Spatial scale encompasses global distribution ranges of sampled Artemisia species and allied taxa.
Data exclusions	We excluded nuclear gene markers with species coverage below 50% or aligned sequence length shorter than 150 base pairs from phylogenetic analysis.
Reproducibility	Reproducibility is ensured by depositing all data, results, and analysis scripts in GenBank, figshare (https://doi.org/10.6084/m9.figshare.28164335), and Supplementary files, with detailed methodologies provided in the Methods section. All experimental and analytical procedures were successfully repeated, with no failed attempts
Randomization	Randomization was not applicable to our study, as our goal was to maximize the number of Artemisia species included in the analyses.
Blinding	Does not apply in a study of this type.

Did the study involve field work? ☐ Yes ☒ No

Reporting for specific materials, systems and methods

We require information from authors about some types of materials, experimental systems and methods used in many studies. Here, indicate whether each material, system or method listed is relevant to your study. If you are not sure if a list item applies to your research, read the appropriate section before selecting a response.

Materials & experimental systems

n/a	Involved in the study	
☒	☐	Antibodies
☒	☐	Eukaryotic cell lines
☒	☐	Palaeontology and archaeology
☒	☐	Animals and other organisms
☒	☐	Clinical data
☒	☐	Dual use research of concern
☐	☒	Plants

Methods

n/a	Involved in the study	
☒	☐	ChIP-seq
☒	☐	Flow cytometry
☒	☐	MRI-based neuroimaging

Dual use research of concern

Policy information about dual use research of concern

Hazards

Could the accidental, deliberate or reckless misuse of agents or technologies generated in the work, or the application of information presented in the manuscript, pose a threat to:

No	Yes	
☒	☐	Public health
☒	☐	National security
☒	☐	Crops and/or livestock
☒	☐	Ecosystems
☒	☐	Any other significant area

Experiments of concern

Does the work involve any of these experiments of concern:

No	Yes	
☒	☐	Demonstrate how to render a vaccine ineffective
☒	☐	Confer resistance to therapeutically useful antibiotics or antiviral agents
☒	☐	Enhance the virulence of a pathogen or render a nonpathogen virulent
☒	☐	Increase transmissibility of a pathogen
☒	☐	Alter the host range of a pathogen
☒	☐	Enable evasion of diagnostic/detection modalities
☒	☐	Enable the weaponization of a biological agent or toxin
☒	☐	Any other potentially harmful combination of experiments and agents

Plants

Seed stocks	Not applicable to this study
Novel plant genotypes	Not applicable to this study
Authentication	Not applicable to this study
